# The key micronutrient copper orchestrates broad-spectrum virus resistance in rice

**DOI:** 10.1126/sciadv.abm0660

**Published:** 2022-07-01

**Authors:** Shengze Yao, Jinrui Kang, Ge Guo, Zhirui Yang, Yu Huang, Ying Lan, Tong Zhou, Liying Wang, Chunhong Wei, Zhihong Xu, Yi Li

**Affiliations:** 1 The State Key Laboratory of Protein and Plant Gene Research, School of Life Sciences, Peking University, Beijing 100871, China.; 2 Institute of Plant Protection, Jiangsu Academy of Agricultural Sciences, Nanjing 210014, China.

## Abstract

Copper is a critical regulator of plant growth and development. However, the mechanisms by which copper responds to virus invasion are unclear. We previously showed that SPL9-mediated transcriptional activation of *miR528* adds a previously unidentified regulatory layer to the established ARGONAUTE (AGO18)–miR528–*L-ascorbate oxidase* (*AO*) antiviral defense. Here, we report that rice promotes copper accumulation in shoots by inducing copper transporter genes, including *HMA5* and *COPT*, to counteract viral infection. Copper suppresses the transcriptional activation of *miR528* by inhibiting the protein level of SPL9, thus alleviating miR528-mediated cleavage of *AO* transcripts to strengthen the antiviral response. Loss-of-function mutations in *HMA5*, *COPT1*, and *COPT5* caused a significant reduction in copper accumulation and plant viral resistance because of the increased SPL9-mediated *miR528* transcription. Gain in viral susceptibility was mitigated when *SPL9* was mutated in the *hma5* mutant background. Our study elucidates the molecular mechanisms and regulatory networks of copper homeostasis and the SPL9-miR528-AO antiviral pathway.

## Teaser

Copper, an essential micronutrient, confers rice resistance to virus infection by inhibiting SPL9-mediated *miR528* transcription.

## INTRODUCTION

Rice (*Oryza sativa*) is a major food supply for more than half of the world’s population (*1*, *2*). Rice production is limited by many factors, including viral pathogens that cause substantial yield and quality losses, posing a constant threat to global food security (*2*–*5*). Breeding rice cultivars resistant to viruses by co-opting the host antiviral defense mechanisms is perhaps the most effective and environmentally friendly measure to meet this challenge. However, our limited understanding of virus-host interactions has hampered this effort. *Rice stripe virus* (RSV; with a genome comprising four negative-stranded RNAs named RNA1–4) is a highly destructive pathogen that belongs to the *Tenuivirus* genus in the Phenuiviridae family and is transmitted by the small brown planthopper (*Laodelphax striatellus*) in a propagative manner, as even the progeny from viruliferous (carrying the virus) female adults can transmit the virus to rice seedlings (*2*, *6*, *7*).

Current studies on virus-host interactions in model dicotyledonous plants have revealed how plants have developed multiple layered immune systems in the arms race between virus and host, including RNA silencing, dominant nucleotide-binding leucine-rich repeat (NLR) resistance genes (*R-*genes), recessive resistance genes [eukaryotic translation initiation factors (eIFs)], phytohormone signaling, and reactive oxygen species (ROS) (*2*, *5*). Plants orchestrate these complex processes to defend themselves against different viral infections (*8*–*15*). However, the antiviral defense network is not identical in monocotyledonous and dicotyledonous plants: For example, to date, *R*-genes such as NLRs do not appear to play a major role in rice antiviral defense (*11*, *16*). Our previous work and that of others have demonstrated that the phytohormone jasmonic acid (JA) accumulates in response to RSV infection and induces *AGO18* transcription through the previously established JAMYB transcriptional regulation pathway (*17*). JA-induced *JAMYB* transcription in turn stimulates *AGO18* expression, leading to the sequestration by AGO18 of the two microRNAs (miRNAs) miR168 and miR528, which normally negatively regulate their targets *AGO1* and *L-ascorbate oxidase* (*AO*) (*6*, *7*, *17*). Here, AGO18 acts as a decoy, whereby miR168 and miR528 are loaded into AGO18, thus preventing their binding to their targets, *AGO1* and *AO*. AGO1 binds viral short interfering RNAs (siRNAs) to cleave viral genomic RNAs through the RNA-induced silencing complex (RISC), while AO elevates the basal ROS levels to restrict viral infection (*6*, *7*). miR168 and miR528 are two of the most abundant miRNAs in rice, and miR528 is a monocot-specific miRNA (*6*, *7*). We further demonstrated that SQUAMOSA promoter-binding–like protein 9 (SPL9) regulates the transcription of *miR528*; *SPL9* itself is regulated by RSV infection (*18*). In addition, several AUXIN RESPONSE FACTORs (ARFs) from the auxin pathway were shown to promote ROS accumulation, which complements other identified pathways involved in rice resistance to different viral infections (*19*–*21*).

Copper is an essential micronutrient that plays a critical role in multiple physiological processes in plants, such as iron mobilization, protein transport, cell wall metabolism, mitochondrial respiration, photosynthetic electron transfer, and phytohormone signaling (*22*–*24*). Copper also triggers ROS accumulation through oxidation-reduction reactions, and copper-binding proteins (usually antioxidant enzymes) eliminate ROS to maintain copper homeostasis (*25*, *26*). While some copper is necessary for plants to thrive, too much (excess) or too little (deficiency) copper constitutes an abiotic stress that severely limits plant development (*22*).

In plants, copper ions are mainly transported by the copper transporter (COPT) family, the cation transport HEAVY METAL adenosine triphosphatase P-type (HMA) family, the yellow stripe–like (YSL) transporter family, and the ZINC/IRON-REGULATED TRANSPORTER–LIKE PROTEIN (ZIP) (*26*–*31*). The rice genome encodes seven COPTs, one HMA-type transporter OsHMA5, and one YSL-type transporter YSL16, which contribute to copper homeostasis by regulating copper uptake and transport (*27*, *32*, *33*). Arabidopsis SPL7 (AtSPL7) and its ortholog Chlamydomonas CRR1 (CrCRR1) modulate copper homeostasis through transcriptionally regulating copper miRNAs, such as miR398, miR408, miR397, and miR857 (*34*–*40*). COPT genes such as *AtCOPT1* and *AtCOPT6*, as well as other genes, are also transcriptionally controlled by AtSPL7 to maintain copper homeostasis (*26*, *37*, *41*). Although copper has been used to protect against pathogens for more than 100 years, such as the application of the copper-based Bordeaux mixture since the 1890s (*42*, *43*), and confers resistance to the pathogenic bacterium *Xanthomonas oryzae* pv. *oryzae* (Xoo) through COPT1 and COPT5 in rice (*42*), the molecular mechanisms underlying how copper signaling regulates plant antiviral defense are unclear.

In this study, we show that RSV infection induces the expression of copper transporter genes including *HMA5*, *COPT1*, and *COPT5* to promote the accumulation of copper in shoots and that high copper levels in shoots enhance rice antiviral defense. Mutants in HMA5, COPT1, and COPT5 accumulated less copper in their shoots and exhibited a compromised resistance to virus infection. Exogenous copper increased rice antiviral defenses by blocking the transcriptional up-regulation of *miR528* by SPL9. Furthermore, elevating copper content in vitro or in vivo promoted rice antiviral defense by regulating SPL9-miR528-AO–mediated ROS signaling. Notably, copper regulated rice antiviral defense in a broad-spectrum manner. Our study elucidates the molecular mechanisms and regulatory network of copper homeostasis and the SPL9-miR528-AO antiviral pathway.

## RESULTS

### Viral infection alters the expression of copper-related genes, and copper orchestrates rice antiviral immune response in general

**Fig. 1.**
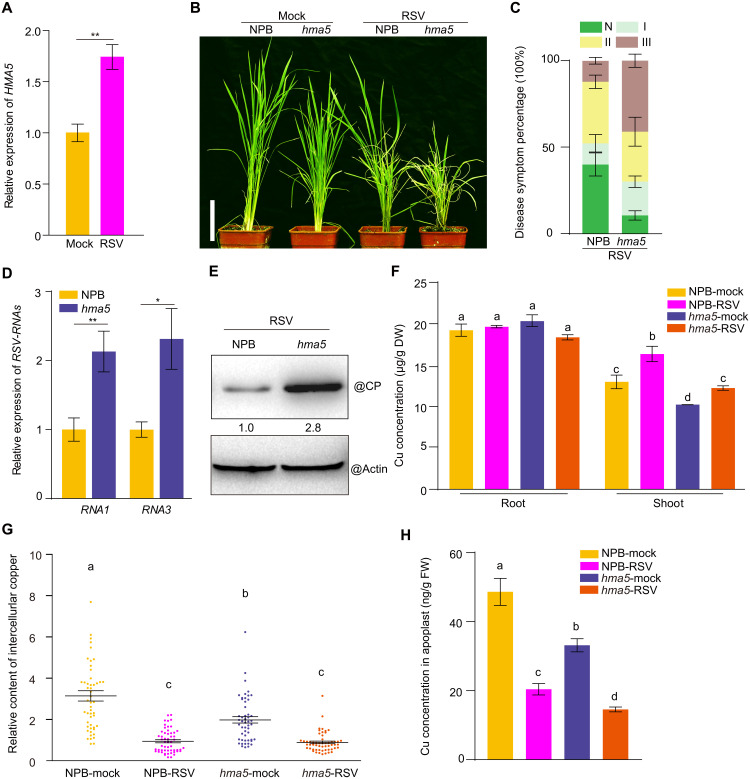
Copper regulates the rice antiviral immune response, and viral infection changes copper location in rice. (**A**) Reverse transcription quantitative polymerase chain reaction (RT-qPCR) analysis of *HMA5* transcript levels in the mock-inoculated or RSV-infected Nipponbare (NPB). (**B**) Symptoms of mock-inoculated or RSV-infected wild-type NPB and *hma5* mutant at 4 wpi (weeks post inoculation). Scale bar, 10 cm. (**C**) Percentages of disease symptom grades between RSV-infected NPB and *hma5*. (**D**) RT-qPCR analysis of *RSV-RNA1* and *RSV-RNA3* transcript levels in RSV-infected NPB and *hma5*. (**E**) RSV coat protein (CP) abundance in RSV-infected NPB and *hma5* by immunoblotting. Actin was used as loading control. (**F**) Copper concentration in shoot and root of mock-inoculated or RSV-infected NPB and *hma5*, as measured by inductively coupled plasma optical emission spectrometry (ICP-OES). DW, dry weight. (**G**) Relative copper content of the intercellular space (shown in fig. S4D) detected by energy-dispersive spectroscopy (EDS) in mock-inoculated or RSV-infected NPB and *hma5*. Each dot represents one intercellular space. (**H**) Copper concentration in the apoplasm of mock-inoculated or RSV-infected NPB and *hma5*, as measured by ICP-OES. FW, fresh weight. Data are shown as means ± SD (*n* = 3 biological repeats). Asterisks mark significant differences according to Student’s *t* test: *P ≤ 0.05; **P ≤ 0.01; Tukey’s test was performed for multiple comparisons; different letters indicate significant differences between the compared pairs (*P* ≤ 0.05).

We used the rice RSV system to explore the fundamental role of copper in rice antiviral responses. We first analyzed gene expression profiles through transcriptome deep RNA sequencing (RNA-seq) of mock-control and RSV-infected rice seedlings (*17*). The expression of over 3000 genes significantly changed upon viral infection (*17*); among them, we identified approximately one-third of the 227 copper-responsive genes (genes induced or inhibited by copper treatment) that were previously identified by microarray assays (fig. S1, A and B) (*44*). More specifically, we noticed several copper-related genes, encoding copper transporters and copper-binding proteins, whose expression was induced by viral infection (Fig. 1A and fig. S1, C and D). We therefore wondered if these copper-related genes might contribute to rice antiviral defenses. To answer this question, we used the *hma5* mutant, which contains a loss-of-function mutation in the HMA5 (*Tos17* insertion) (fig. S2, A to C) resulting in a defect in copper transport from the root to the shoot (*32*). We challenged the *hma5* mutant with RSV infection by inoculation via viruliferous small brown planthoppers. The wild-type Nipponbare (NPB) was used as a control. We scored RSV-infected rice plants according to the severity of disease symptoms (fig. S2D). Compared to NPB, *hma5* mutant seedlings were more susceptible to RSV infection and exhibited a higher percentage of severe disease symptoms (Fig. 1, B and C). We assessed the extent of RSV replication in planta by measuring the levels of the RSV genome segment *RNA3* by reverse transcription quantitative polymerase chain reaction (RT-qPCR) and the abundance of the RSV coat protein (CP) by immunoblotting. RSV *RNA3* and CP both accumulated to a much higher level in the *hma5* mutant than in NPB (Fig. 1, D and E). For comparison, we also analyzed RNA-seq data for rice seedlings infected with *Rice dwarf virus* (RDV) (*45*), a double-stranded RNA virus from the *Phytoreovirus* genus (*3*). Similar changes in copper-related genes were observed in RDV-infected rice plants (fig. S3, A to D). Although RNA-seq data showed that *HMA5* transcription does not change obviously upon RDV infection (fig. S3E), we were curious to know whether the lower copper concentration in the *hma5* mutant also affected rice resistance to RDV infection, prompting us to challenge NPB and *hma5* by RDV. We determined that *hma5* also displays much more severe disease symptoms, with a greater accumulation of RDV genome segment S2 and CP (P2) compared to NPB (fig. S3, F to H). Thus, the lower copper levels in rice shoots generally compromised resistance against virus infection.

### Copper location changes significantly upon viral infection

Given that *HMA5* expression was up-regulated upon RSV infection (Fig. 1A) and that the *hma5* mutant was more susceptible to RSV infection, we next asked whether virus infection altered the accumulation and location of copper. To this end, we first determined the copper concentration of mock-inoculated (mock) and RSV-infected (RSV) wild-type rice NPB plants. Rice leaves were dried and digested in concentrated nitric acid before measuring their copper concentration by inductively coupled plasma optical emission spectrometry (ICP-OES) (*32*). Compared to healthy mock-inoculated rice, virus infection caused a marked increase in shoot copper levels of up to 25%, while we observed no obvious change in the root (Fig. 1F). Similar results were also obtained by detecting relative copper content in mock-inoculated and RSV-infected NPB leaves through x-ray fluorescence spectroscopy (XRF), a commonly used method for detecting ion distribution in plant tissue (fig. S4, A to C) (*46*). In parallel, to define the distribution of copper in plant tissues upon RSV infection, rice leaves were ultrasectioned and observed by transmission electron microscopy (TEM), in conjunction with measurements of relative copper content by energy-dispersive spectroscopy (EDS) (Fig. 1G and fig. S4D) (*46*). Unexpectedly, although copper content increased in the shoot upon virus infection, copper levels decreased in the intercellular space of both NPB and *hma5* (Fig. 1G), indicating that copper gathers more inside of cells during virus infection. This conclusion was further confirmed by extracting the leaf apoplasm (*47*) and measuring its copper concentration by ICP-OES (Fig. 1H). Furthermore, we examined the distribution of copper in subcellular organelles such as nuclei, mitochondrion, and chloroplast by TEM-EDS and ICP-OES and found that the relative copper content was significantly reduced in chloroplasts and intercellular space but increased in intracellular space upon viral infection, which further indicated that virus infection would lead to the accumulation of copper ions in the intracellular space (fig. S4, E and F). These results suggest that RSV infection alters the content and location of copper in rice plants and that copper orchestrates the plant antiviral immune response.

### Copper regulates the SPL9-miR528-AO pathway

**Fig. 2.**
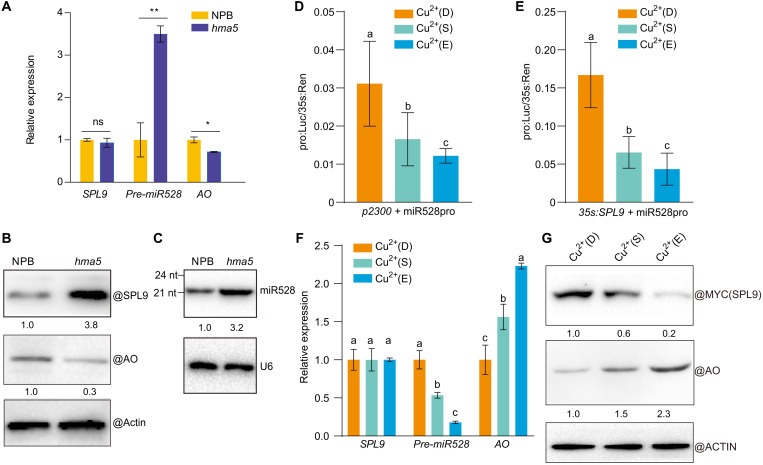
Copper regulates the SPL9-miR528-AO pathway. (**A**) RT-qPCR analysis of the relative transcript levels of *SPL9*, *pre-miR528*, and *AO* in NPB and *hma5*. (**B**) Detection of SPL9 and AO protein abundance in NPB and *hma5* by immunoblotting. Actin was used as loading control. (**C**) Detection of miR528 in NPB and *hma5* by Northern blot. U6 was used as loading control. (**D** and **E**) Luciferase assay of the transcriptional activity of SPL9 on *miR528* transcription in rice protoplasts. Different construct combinations (pCAMBIA2300, pCAMBIA2300-MYC-SPL9, and pGreenII0800:miR528 promoter: luciferase) were transfected, and the protoplasts were incubated in conditions of copper deficient (0 μM Cu^2+^, D), copper sufficient (0.5 μM Cu^2+^, S), and copper excess (10 μM Cu^2+^, E). Two days later, luciferase activities were determined. (**F**) RT-qPCR analysis of *SPL9*, *pre-miR528*, and *AO* transcript levels in protoplasts treated by the indicated copper concentrations. (**G**) Detection of SPL9 (MYC-tagged) and AO in protoplasts treated by the indicated copper concentrations by immunoblotting. Actin was used as loading control. The average values (±SD) from three biological repeats are shown. Asterisks mark significant differences according to Student’s *t* test: *P ≤ 0.05; **P ≤ 0.01; ns, no significant difference. Tukey’s test was performed for multiple comparisons; different letters indicate significant differences between the compared pairs (*P* ≤ 0.05).

Copper status regulates the expression of several copper-related genes. The rice transcription factor SQUAMOSA promoter-binding–like protein 9 (OsSPL9) is orthologous to AtSPL7 (fig. S5A), which is a central regulator of copper homeostasis (*36*–*40*). We showed previously that SPL9 activates the transcription of the *miR528* locus, which regulates rice antiviral responses through the miR528-mediated cleavage of *AO* transcripts (*7*, *18*), the encoded AO protein being a copper-binding protein (fig. S5B). We proposed that copper may orchestrate rice antiviral defenses through the SPL9-miR528-AO pathway. To specifically test this hypothesis, we measured the relative transcript levels of *SPL9*, *pre-miR528*, and *AO* in the *hma5* mutant by RT-qPCR, as well as SPL9 and AO abundance by immunoblotting. SPL9 protein abundance, rather than *SPL9* transcript levels, increased in the *hma5* mutant relative to NPB. The accumulation of SPL9 resulted in the activation of *miR528* transcription, hence reducing the abundance of AO (Fig. 2, A to C). To further validate that the SPL9-miR528-AO pathway is regulated by copper, we used the dual-luciferase reporter system. We cotransfected a construct encoding MYC-tagged SPL9 and a reporter construct consisting of the *miR528* promoter driving *luciferase* in rice protoplasts prepared from NPB plants. The protoplasts were then treated with 0, 0.5, or 10 μM CuSO_4_ to simulate copper-deficient, copper-sufficient, and copper-excess conditions, respectively. Compared to copper sufficiency, copper deficiency raised SPL9 abundance and the transcriptional regulation of *miR528*, thus reducing AO levels, whereas copper excess repressed this process (Fig. 2, D to G). Last, to test the direct interaction between SPL9 and copper ions, we performed a microscale thermophoresis (MST) assay (*18*, *45*). Accordingly, we purified recombinant SPL9 DNA-binding domain (SPL9 SBP, ca. 33 kDa) from *Escherichia coli* and mixed SPL9 SBP with a range of copper or calcium concentrations: SPL9 SBP bound to the copper ions directly but did not bind to calcium ions (fig. S6A). Furthermore, the binding of SPL9 SBP to the *miR528* promoter harboring GTAC motifs was impaired by copper ions but not by calcium ions, as evidenced by electrophoretic mobility shift assays (EMSAs) (fig. S6, B and C). These results suggest that copper regulates the SPL9-miR528-AO pathway.

### Regulation of antiviral defense by copper requires SPL9

**Fig. 3.**
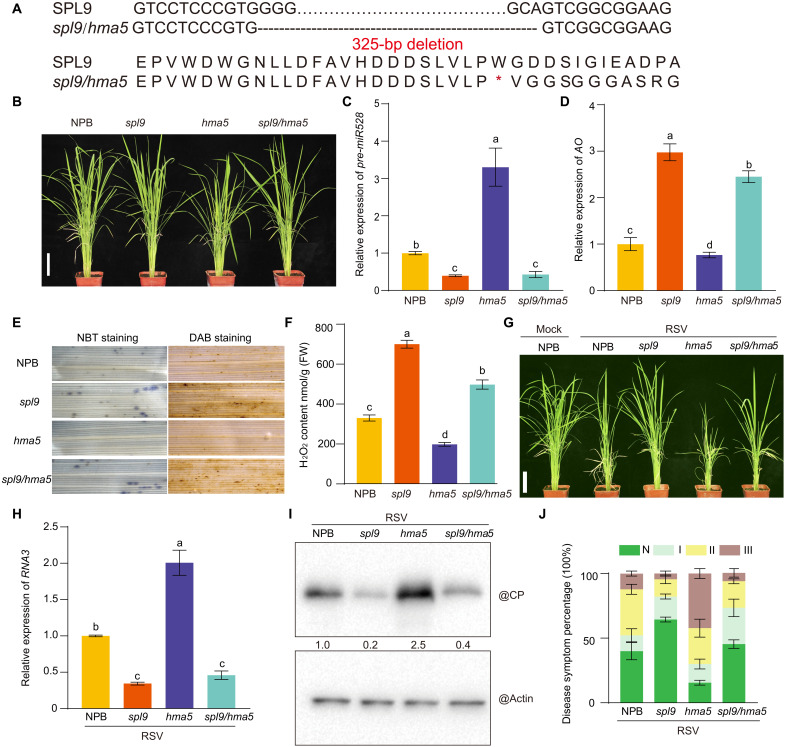
Copper regulates rice antiviral immune response via SPL9. (**A**) Construction of the *spl9/hma5* double mutant in the *hma5* background using CRISPR-Cas9–mediated genome editing. The DNA sequences of the *spl9* mutant are shown and consist of a 325–base pair (bp) deletion resulting in a premature termination of translation. (**B**) Phenotypic comparison of NPB, *spl9*, *hma5*, and *spl9/hma5* plants at 6 weeks after germination. Scale bar, 10 cm. (**C** and **D**) RT-qPCR analysis of *pre-miR528* (C) and *AO* (D) transcript levels in the indicated genotypes. (**E**) In situ detection of ROS accumulation in rice leaves using 3,3′-diaminobenzidine (DAB) and nitroblue tetrazolium (NBT) staining. (**F**) Quantification of H_2_O_2_ accumulation in rice leaves of the indicated genotypes. (**G**) Symptoms of mock-inoculated NPB and RSV-infected NPB, *spl9*, *hma5*, and *spl9/hma5* plants at 4 wpi. Scale bar, 10 cm. (**H**) RT-qPCR analysis of *RSV-RNA3* transcript in RSV-infected NPB, *spl9*, *hma5*, and *spl9/hma5* plants. (**I**) Detection of RSV-CP protein in RSV-infected NPB, *spl9*, *hma5*, and *spl9/hma5* plants by immunoblotting. Actin was used as loading control. (**J**) Percentages of disease symptom grades in RSV-infected NPB, *spl9*, *hma5*, and *spl9/hma5* plants. The average values (±SD) from three biological repeats are shown. Tukey’s test was performed for multiple comparisons; different letters indicate significant difference between the compared pairs (*P* ≤ 0.05).

To further explore the antiviral function of copper in regulating the SPL9-miR528-AO pathway, we generated an *spl9/hma5* double mutant by CRISPR-Cas9–mediated genome editing of *SPL9* in the *hma5* mutant background (Fig. 3A). The resulting *spl9/hma5* double mutant displayed almost normal growth and development at the vegetative stage (Fig. 3B and fig. S7, A and B). In contrast to *hma5*, in which *pre-miR528* levels were high, *pre-miR528* accumulated to much lower levels in both the single *spl9* mutant and the *spl9/hma5* double mutant because of the loss of function of SPL9-mediated transcriptional activation (Fig. 3C). In agreement, the *spl9/hma5* double mutant also showed greater abundance of AO and ROS levels in the form of both H_2_O_2_ and O_2_^•−^ compared to the *hma5* mutant (Fig. 3, D to F). We further tested the antiviral responses of the *spl9*, *hma5*, and *spl9/hma5* mutants by inoculating seedlings with RSV, using NPB as a control. Compared to NPB, *hma5* was the most susceptible to infection, as shown by growth retardation and accumulation of RSV RNAs and CP; by contrast, the *spl9* single mutant and the *spl9/hma5* double mutant were more resistant to viral infection than *hma5* and NPB, with the *spl9/hma5* mutant almost displaying the level of virus resistance as *spl9* (Fig. 3, G to J). We had previously shown that AO plays a pivotal role in the up-regulation of the basal ROS levels (*7*, *18*), as an AO loss-of-function mutation exhibited very high susceptibility to viral infection because of a decrease in ROS accumulation (fig. S8, A to I). Since AO is a copper binding protein, we thus tested whether AO-mediated antiviral activity requires its copper binding ability. We therefore generated a mutant version of AO (mAO) that cannot bind to copper, which abrogated not only its enzymatic activity but also its antiviral properties (fig. S8, A to I). Thus, these results further suggest that copper relies on the SPL9-miR528-AO pathway to confer antiviral defense, although they do not exclude additional contributing pathways.

### Exogenous application of copper ions enhances rice resistance to RSV infection

**Fig. 4.**
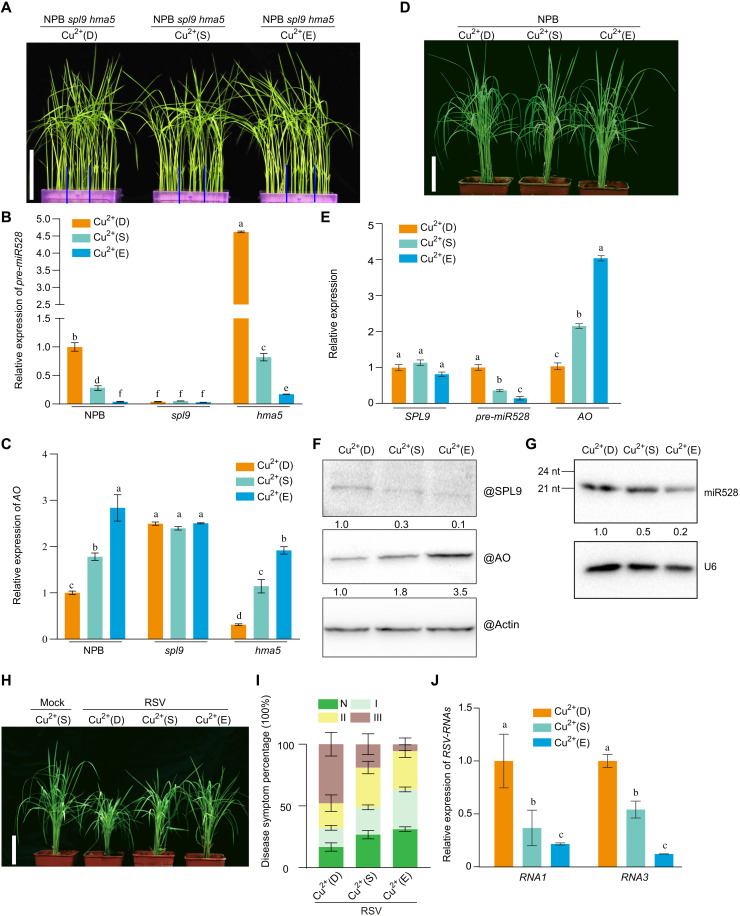
Exogenous application of copper enhances rice resistance to virus infection. (**A**) Representative photographs of NPB, *spl9*, and *hma5* plants treated by the indicated exogenous copper concentrations at 6 weeks after germination. Scale bar, 10 cm. The blue lines indicate the separation between the different rice plants. (**B** and **C**) RT-qPCR analysis of *pre-miR528* (B) and *AO* (C) transcript levels in the indicated genotypes. (**D**) Representative photographs of NPB treated by the indicated exogenous copper concentrations at 6 weeks after germination. Scale bar, 10 cm. (**E**) RT-qPCR analysis of *SPL9*, *pre-miR528*, and *AO* transcript levels in NPB exposed to various copper concentrations. (**F**) Immunodetection of SPL9 and AO protein levels in NPB exposed to various copper concentrations. Actin was used as loading control. (**G**) Detection of miR528 in NPB exposed to various copper concentrations by Northern blot. U6 was used as loading control. nt, nucleotide. (**H**) Symptoms of mock-inoculated or RSV-infected NPB plants treated by the indicated exogenous copper concentrations at 4 wpi. Scale bar, 10 cm. (**I**) Percentage of disease symptom grades in RSV-infected NPB treated by the indicated exogenous copper concentrations. (**J**) RT-qPCR analysis of *RSV-RNAs* transcript levels as a function of exogenous copper concentrations. The average values (±SD) from three biological repeats are shown. For each gene, Tukey’s test was performed for multiple comparisons; different letters indicate significant differences between the compared pairs (*P* ≤ 0.05).

Given that SPL9 binding to *miR528* promoter was regulated by copper in rice protoplasts, we wished to assess whether exogenous copper applications to rice seedlings might have the same effect. To answer this question, we first exposed NPB, *spl9*, and *hma5* seedlings to three copper nutritional states: deficient (0 μM CuSO_4_), sufficient (0.5 μM CuSO_4_), and excess (10 μM CuSO_4_). All genotypes showed the same growth phenotypes at the vegetative stage (Fig. 4A and fig. S9). We then analyzed molecular phenotypes under these conditions by measuring *SPL9*, *pre-miR528*, and *AO* transcript levels by RT-qPCR. In the *spl9* mutant, *pre-miR528* levels remained low, while *AO* transcript levels accumulated to high levels at all copper concentrations because of the absence of SPL9 (Fig. 4, B and C). We also treated NPB rice seedlings with the same range of copper concentrations. SPL9 protein abundance substantially decreased and progressively lost the ability to activate *miR528* transcription with higher copper levels, which was accompanied by a gradual rise in *AO* transcripts (Fig. 4, D to G). To determine whether the copper status affected plant responses to viral infection, we challenged rice seedlings growing under the three copper concentrations with RSV infection. We found that copper-excess seedlings experienced less severe stunting of their growth after RSV infection than copper-sufficient seedlings, whereas copper-deficient seedlings exhibited more severe stunting (Fig. 4, H and I). Consistent with these observations, the abundance of RSV RNAs decreased with higher copper concentrations (Fig. 4J). Our results thus demonstrate the positive role of copper during rice antiviral defense.

### The regulation of SPL9 by copper is specific

The rice genome encodes 19 *SPL* members, prompting us to ask whether the regulation of SPL9 by copper was specific. To determine this, we measured the transcript levels of all other *SPL*s in rice seedlings grown under copper-deficient, copper-sufficient, or copper-excess conditions. We also assessed the expression level of genes downstream of SPL6, SPL10, SPL14, and SPL17, such as *INOSITOL REQUIRING1* (*IRE1*), *Hairy Leaf 6* (*HL6*), *WRKY45*, and *PIN-FORMED 1b* (*PINIb*), respectively (*48*–*51*). In all cases, copper did not show any effect on the expression of other *SPL* members or their downstream targets (fig. S10). Therefore, we conclude that copper regulation of SPL9 is specific.

### Copper transporters contribute differently to the copper homeostasis and antiviral response

**Fig. 5.**
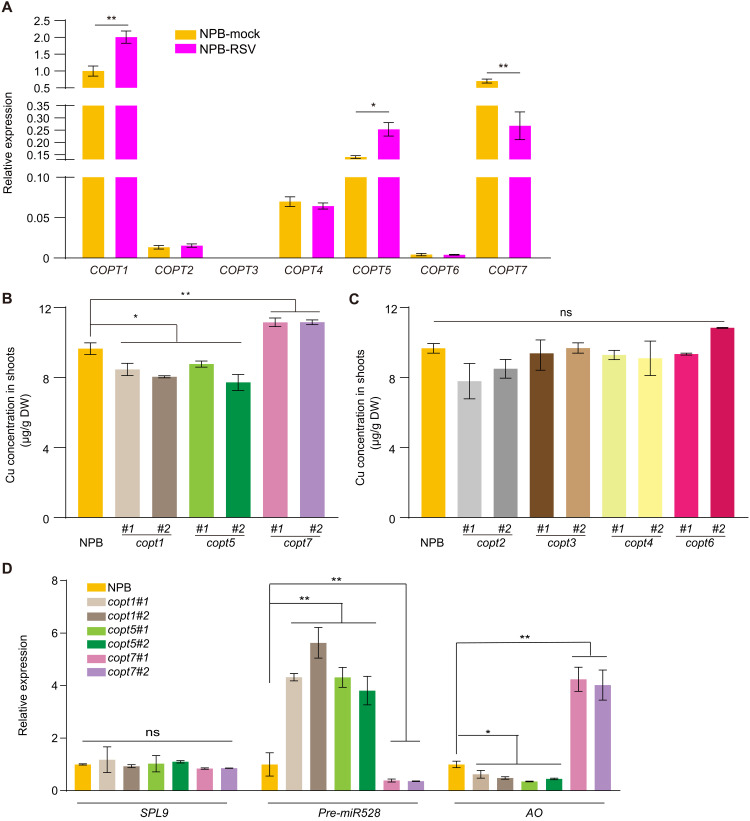
Response of *COPT* family members to virus infection and Cu accumulation. (**A**) RT-qPCR analysis of *COPT1*–*7* transcript levels in mock-inoculated or RSV-infected NPB plants. (**B**) Copper concentration in the shoots of NPB and *copt1*, *copt5*, and *copt7* plants, as measured by ICP-OES. (**C**) Copper concentration in the shoots of NPB and *copt2*, *copt3*, *copt4*, and *copt6* plants, as measured by ICP-OES. (**D**) RT-qPCR analysis of *SPL9*, *pre-miR528*, and *AO* transcript levels in NPB and *copt1*, *copt5*, and *copt7* plants. The average values (±SD) from three biological repeats are shown. Asterisks mark significant differences according to Student’s *t* test: *P ≤ 0.05; **P ≤ 0.01.

**Fig. 6.**
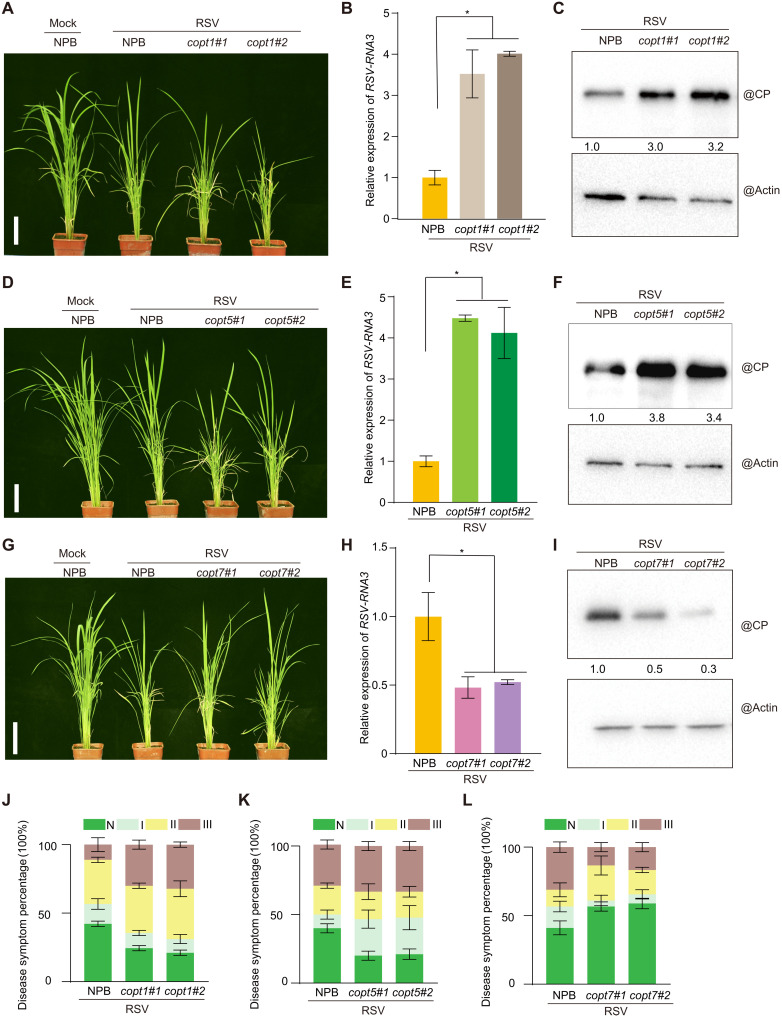
Copper transporters exert different regulation on virus infection. (**A**) Symptoms of mock-inoculated NPB and RSV-infected NPB and *copt1* plants at 4 wpi. Scale bar, 10 cm. (**B**) RT-qPCR analysis of *RSV-RNA3* transcript levels in RSV-infected NPB and *copt1*. (**C**) Immunodetection of RSV-CP protein in RSV-infected NPB and *copt1* plants. Actin was used as loading control. (**D**) Symptoms of mock-inoculated NPB and RSV-infected NPB and *copt5* plants at 4 wpi. Scale bar, 10 cm. (**E**) RT-qPCR analysis of *RSV-RNA3* transcript levels in RSV-infected NPB and *copt5*. (**F**) Detection of RSV-CP protein in RSV-infected NPB and *copt5* plants by immunoblotting. Actin was used as loading control. (**G**) Symptoms of mock-inoculated NPB and RSV-infected NPB and *copt7* plants at 4 wpi. Scale bar, 10 cm. (**H**) RT-qPCR analysis of the *RSV-RNA3* transcript levels in RSV-infected NPB and *copt7*. (**I**) Immunodetection of RSV-CP protein in RSV-infected NPB and *copt7* plants. Actin was used as loading control. (**J** to **L**) Percentages of disease symptom grades in RSV-infected NPB, *copt1* (J), *copt5* (K), and *copt7* (L) mutant lines. The average values (±SD) from three biological repeats are shown. Asterisks mark significant differences according to Student’s *t* test: *P ≤ 0.05.

COPTs play key roles in copper homeostasis in diverse species such as yeast (*Saccharomyces cerevisiae*), humans, and plants. To elucidate the functions of the seven rice COPTs (fig. S11A) in response to viral infection, we first analyzed *COPT* expression levels upon viral infection by RT-qPCR. Each *COPT* gene showed a different expression pattern upon virus infection, with *COPT1* and *COPT5* strongly up-regulated and *COPT7* down-regulated, while *COPT2*, *COPT3*, *COPT4*, and *COPT6* transcript levels were comparable between control and RSV-infected seedlings (Fig. 5A). To further understand the functions of *COPTs* in copper accumulation and antiviral responses, we generated individual *copt* mutants using CRISPR-Cas9–mediated genome editing in the NPB background (fig. S11B), which showed no visible phenotypes at the vegetative stage under normal growth conditions (fig. S11C). However, the *copt1* and *copt5* mutants did accumulate less copper than NPB in their shoots, in agreement with a previous report (*42*), while *copt7* mutant lines had more copper in shoots (Fig. 5B). The other single *copt* mutant lines did not significantly alter shoot copper concentration (Fig. 5C). We then asked whether the variation in copper concentration seen in *copt1*, *copt5*, and *copt7* mutant lines also affected the SPL9-miR528-AO module and rice resistance to RSV infection. Similar to the *hma5* mutant, *copt1* and *copt5* mutants displayed higher *pre-miR528* transcript levels than NPB (Fig. 5D). Conversely, *pre-miR528* was down-regulated in *copt7* mutant lines compared to NPB because of copper-imposed repression of SPL9 transcriptional activity (Fig. 5D). Consequently, *AO* transcript levels were negatively correlated with *pre-miR528* levels, with lower *AO* levels in *copt1* and *copt5* mutant lines and higher levels in *copt7*. Consistent with the above results, RSV infection assays showed that *COPT1* and *COPT5* promote resistance to virus infection, while *COPT7* restricted virus infection (Fig. 6). Thus, our results clearly demonstrate the diversified function of COPT family members on copper accumulation and antiviral defense and further substantiate the role of copper in viral resistance through manipulating the SPL9-miR528-AO pathway.

## DISCUSSION

Rice provides food for over half of the world’s population, but production and yields are periodically threatened by diseases caused by viral pathogens (*2*–*4*). As a model monocotyledonous crop, rice defenses against viral infections are not identical to those of dicotyledonous plants like Arabidopsis. No *NBS-LRR–like* resistance genes have been identified whose encoded proteins specifically recognize viral avirulence (Avr) proteins in rice. Our previous studies and those of other groups revealed that at least three pathways regulate rice antiviral defenses. First, JA enhances RNA interference through OsAGO18 as a core factor and is one of the most important and effective antiviral defense pathways in rice (*6*, *7*, *17*). *OsAGO18* transcription is positively regulated by the JA-responsive transcription factor JAMYB upon viral infection. Overexpressing the virus CP gene (*CP*) activates the JA signaling pathway and up-regulates *OsAGO18* expression through JAMYB (*17*). OsAGO18 then competitively associates with miR168 and miR528 to prevent them from binding to transcripts for their targets, *OsAGO1* and *AO*, respectively. OsAGO1 further mediates antiviral defense through the virus-derived siRNA pathway (*6*). Second, viral infection down-regulates the transcription of *miR528* by decreasing the abundance of SPL9, the transcription factor activating *miR528* transcription (*18*), although *SPL9* transcription is not affected. Third, other plant hormones like auxin and brassinosteroids are also involved in rice responses to viral infection. The ARF family of transcription factors within the auxin pathway differentially regulates rice resistance to virus infections (*16*, *19*–*21*). Recently, cross-talk between JA and brassinosteroids was shown to positively regulate rice defense against RSV (*13*).

In this study, we described how copper, an essential micronutrient for plant growth and development, also orchestrates antiviral defenses by regulating the SPL9-miR528-AO pathway. First, viral infection modifies copper location and levels in shoots. Second, viral infection up-regulates the transcription of several copper transporter genes like *HMA5*, *COPT1*, and *COPT5*. *hma5*, *copt1*, and *copt5* loss-of-function mutants accumulated less copper in their shoots and exhibited compromised rice resistance to virus infection. Third, exogenous applications of copper enhanced rice resistance to RSV infection. We therefore propose that copper regulates rice antiviral defense through the SPL9-miR528-AO pathway and functions in a broad-spectrum manner.

**Fig. 7.**
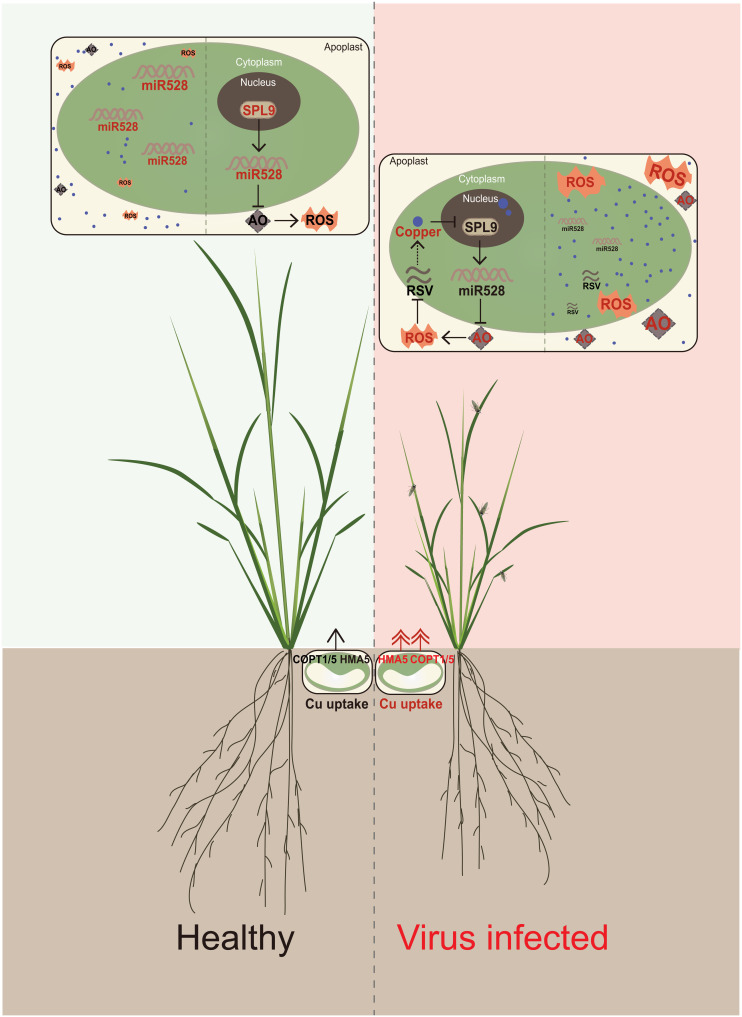
Proposed model for copper participation in antiviral defense through the SPL9-miR528-AO pathway during RSV infection. In healthy rice plants, SPL9 activates the transcription of miR528 and thus represses expression of AO to maintain ROS to an appropriate level. However, when RSV infects rice, the copper transporter genes (e.g., *HMA5*, *COPT1*, and *COPT5*) are induced and copper content increases in the cell, repressing SPL9-mediated *miR528* transcriptional activation. The accumulation of the antiviral factor AO then increases, enhancing the resistance to the virus by increasing ROS accumulation.

We determined the copper content of healthy and RSV-infected rice seedlings by XRF, ICP-OES, and TEM-EDS. We found that copper accumulated in the intracellular space upon viral infection (Fig. 1), a change that may be mediated by adjusting the expression of COPTs (Fig. 5). Copper relocalization upon virus infection may be a key regulatory step in host antiviral defense. We propose that two different processes might take place during viral infection. First, the host might transport more copper from the intercellular space inside the cell to suppress the transcription activity of SPL9. Meanwhile, the virus might somehow block the entry of copper into the cell to counter the copper-triggered defense. We then turned to a genetic dissection of copper concentration with mutants in various copper transporters (e.g., HMA5 and COPTs) and to exogenous applications of copper, which revealed that elevated copper concentration represses *SPL9* transcription and consequently inhibits *miR528* transcription, thus promoting AO-mediated accumulation of ROS. These ROS triggered by copper enhance rice antiviral defense (Fig. 7).

The essential micronutrient copper is involved in an array of biological processes in plants. Here, we found that copper plays an important role in plant antiviral immunity. Both cupric (Cu^2+^) and cuprous (Cu^+^) copper ions are associated with the formation and elimination of ROS (*22*). Copper functions as a cofactor for many enzymes to help maintain their structural and catalytic properties including cytochrome *c*, copper-zinc superoxidase dismutase (CuSOD), as well as AO, which participate in the elimination or formation of ROS. From our research, SPL9 and copper are part of a complex regulatory module that maintains copper homeostasis while also allowing quick responses to biotic or abiotic stress. Copper directly binds to SPL9 to block SPL9 binding to the promoter of its target gene *miR528* (Fig. 2). Exogenous copper treatment and RSV infection also reduce SPL9 abundance without affecting *SPL9* transcription. How *SPL9* transcription is regulated and how copper treatment and RSV infection reduce SPL9 protein levels will be the focus of future investigations.

Mutating *SPL9* in the *hma5* mutant background largely suppressed the severe susceptibility phenotype of the *hma5* mutant, although not completely (Fig. 3), suggesting that other pathway(s) may be involved in copper-mediated antiviral response. Since RNA interference and phytohormone signaling pathways have been reported to participate in antiviral immunity (*6*, *17*–*21*), whether copper also regulates other antiviral pathways and whether antiviral pathways interact with the copper-mediated SPL9 pathway are open questions.

In Arabidopsis, copper deficiency induces the expression of JA biosynthetic genes in flowers and increases endogenous JA accumulation in leaves; these processes are dependent on AtSPL7 (*52*). In rice, we found that RSV infection induces JA biosynthetic genes and JA accumulation, and JA further activates AGO18 transcription through JAMYB, the key transcription factor in the JA pathway, then triggers AGO18-mediated antiviral defense (*17*). AGO18 is specific to monocotyledonous plants and does not exist in dicotyledonous plants (*6*). Therefore, it seems that there is some difference between OsSPL9 and AtSPL7, although OsSPL9 is an ortholog of AtSPL7. Whether OsSPL9 is involved in the JA pathway during RSV infection or copper treatment deserves further research.

It is interesting that copper regulates SPL9 activity rather than that of other SPLs. Most SPLs are mainly involved in plant growth and development (*48*–*51*), making SPL9 quite unique. Among the 200 currently known SPL proteins, SPL9 and its orthologs including AtSPL7 and CrCRR1 (*34*–*37*), which have been well established in copper homeostasis regulation, are evolutionarily conserved and form a single branch in the SPL family evolutionary tree. Notably, the SPL9 branch is represented by a single ortholog in most species (*53*). We and other groups also showed previously that *miR528* is specifically transcriptionally activated by SPL9 rather than other SPLs in rice (*18*, *54*). All these observations indicate that SPL9 and its orthologs were specifically regulated by copper homeostasis in evolution.

## MATERIALS AND METHODS

### Plant growth and virus inoculation

Rice (*O. sativa* spp*. japonica*) plants were grown and inoculated with RSV or RDV as previously described (*6*). The rice cultivar NPB was used as the wild-type control. Briefly, rice seedlings were grown for 2 weeks in a greenhouse at 28° to 30°C and 60 ± 5% relative humidity. Virus inoculations were then performed with the two viruliferous (carrying RSV or RDV) or virus-free (mock) insects. The insects were removed 2 days later, and the rice seedlings were returned in the greenhouse. The rice plants were photographed, and the whole shoots were harvested for further RT-qPCR, and immunoblotting assays at 4 wpi (weeks post inoculation). The percentage of plants with different disease symptom grades was recorded. The mean number of small brown planthoppers on each rice plant was used as the index of nonpreference shown in table S4. At least 30 rice plants were used for each line.

### Transcriptome deep RNA-seq data analysis

RNA-seq data from rice plants infected with RSV or mock treated, infected with RDV or mock treated, and treated with copper or mock treated (microarray-based transcriptome analysis) were obtained from previous reports (*17*, *44*, *45*). Reads per kilobase of transcript per million mapped reads was used to identify differentially expressed genes (DEGs). Venn diagrams of significant DEGs in RSV-infected versus mock-inoculated and copper-treated versus mock-inoculated NPB were constructed, and the associated physiological processes were classified as previously reported (*44*).

### Copper treatments

Rice seeds were sown and germinated in rice nutrient solution containing 2 mM NH_4_NO_3_, 3 mM KNO_3_, 0.1 mM CaCl_2_, 2 mM KCl, 2 mM Ca(NO_3_)_2_, 2 mM MgSO_4_, 0.6 mM KH_2_PO_4_, 1.5 mM NaCl, 0.05 mM NaFe(III)EDTA, 0.05 mM H_3_BO_3_, 5 μM MnCl_2_, 10 μM ZnSO_4_, 0.5 μM CuSO_4_, and 0.1 μM Na_2_MoO_4_ for cultivation in an artificial climate room. After growth for about 5 to 7 days, the original rice nutrient solution was washed away and replaced with fresh nutrient solution containing 0, 0.5, or 10 μM CuSO_4_ (defining copper-deficient, copper-sufficient, and copper-excess conditions) for 7 days. At least 30 rice plants were used for each line.

### Vector construction and rice transformation

The coding sequence of *SPL9* was amplified from cDNA of NPB using RT-PCR and then cloned into the pCam2300:Actin1:OCS vector to generate the pCam2300:Actin1:MYC-SPL9 construct. The *copt1*–*7* mutants in the NPB background and *spl9* mutant in the *hma5* background were generated using CRISPR-Cas9–mediated genome editing as previously described (*55*). All constructs were transformed into rice using Agrobacterium-mediated (*Agrobacterium tumefaciens*) transformation (BIOGLE Genetech, Hangzhou, China), and genome-edited mutants were validated by sequencing in both directions. All primers used for vector construction are listed in table S1.

### Immunoblotting

Equal weights of rice samples were homogenized in liquid nitrogen, followed by the extraction of total proteins in 2× Laemmli buffer [4% (w/v) SDS, 20% (v/v) glycerol, 10% (v/v) 2-mercaptoethanol, 0.004% (w/v) bromophenol blue, and 0.125 M tris-HCl (pH 6.8)]. Protein samples were then boiled at 95°C for 10 min, and the supernatants were collected by centrifugation for 10 min at 10,000*g* at 4°C and separated by SDS–polyacrylamide gel electrophoresis. Proteins were then transferred to nitrocellulose membranes and detected with antibodies against SPL9 (peptide antibody generated against EADIRELKGYHRR; the antiserum was affinity purified by WuXi AppTec, Shanghai, China), AO (antibody raised against the peptide CDSPEQPEPFRHQYDD; the antiserum was affinity purified by WuXi AppTec, Shanghai, China), MYC (ABclonal, Wuhan, China), RSV-CP (*56*) (from J. Wu and X. Zhou), and Actin (CWBIO, Beijing, China). PageRuler Prestained Protein Ladder (Thermo Fisher Scientific) was used as a molecular weight standard. At least eight rice plants were pooled and used for one biological replicate.

### miRNA Northern blotting

Northern blot analysis for miRNA in rice plants was performed as described before (*57*). Biotin-labeled oligonucleotide probes complementary to miR528 were used for Northern blots. The sequences of the probes are listed in table S1.

### RNA extraction and qPCR analysis

Rice samples (usually ~100 mg) were homogenized in liquid nitrogen, and total RNA was extracted with TRIzol reagent (Thermo Fisher Scientific, Waltham, MA, USA) according to the manufacturer’s instructions. Total RNA was then digested with RQ1 ribonuclease-free deoxyribonuclease (DNase) (Promega, Madison, WI, USA) to remove traces of contaminating genomic DNA. Reverse transcription was performed on 2 μg of DNase-treated RNA with M-MLV Reverse Transcriptase (Promega, Madison, WI, USA) using oligo (dT)_18_ primers and Recombinant RNasin Ribonuclease Inhibitor (Promega). qPCRs were performed using the SYBR Green Real-Time PCR Master Mix (Toyobo, Osaka, Japan) following the manufacturer’s instructions. At least eight rice plants were pooled and used for one biological replicate. *OsEF1a* was used as the internal control. The primers used for qPCR are listed in table S1.

### Determination of copper concentration in rice by ICP-OES

The shoots and roots of rice lines were separately harvested and washed in water and washing buffer (10 mM Na_2_-EDTA and 5 mM CaSO_4_). Samples were dried at 70°C for 7 days and then ground to a powder. For each sample, 100 mg of powder was digested in 2 ml of 70% concentrated HNO_3_ (Sigma-Aldrich) at a temperature of up to 130°C. After dilution with ddH_2_O to contain ~5% HNO_3_, the copper concentration in the solution was determined with an ICP-OES (Prodigy 7, Leeman, USA) on the basis of the characteristic electromagnetic radiation emitted by each atom or ion at the Analytical Instrumentation Center of Peking University (PKUAIC, Beijing, China). Diluted 5% HNO_3_ was used as a negative control, with each sample consisting of at least three to four biological replicates. At least 10 rice plants were pooled and used for one biological replicate.

### Transmission electron microscopy energy-dispersive spectroscopy

Rice leaves grown for 3 to 4 wpi were cut into small pieces (1 mm^2^) and fixed in 4% (w/v) paraformaldehyde and 4% (w/v) glutaraldehyde buffered with 0.1 M phosphate buffer pH 7.4 under vacuum. Leaves were stayed at room temperature for 4 hours and then overnight at 4°C. After rinsing the samples four times with phosphate buffer for 15 min each, leaves were postfixed with 2% (w/v) OsO_4_ and 1.5% (w/v) potassium ferrocyanide for 2 hours at room temperature in the dark. After one more wash in phosphate buffer, the samples were soaked in 2% (w/v) OsO_4_ overnight at 4°C. Following two washes in phosphate buffer for 15 min each and two washes in distilled water for 15 min each, specimens were dehydrated in a graded ethanol series of 30% (v/v) and 50% ethanol for 15 min each, immersed for 1 hour in 1% (w/v) uranyl acetate in 70% ethanol, and then in 85, 95, and, lastly, 100% ethanol (twice) and embedded in Spurr’s resin (SPI Supplies, PA, USA). Resin blocks were sectioned with a diamond knife (ultra 35°, Diatome, Switzerland) using a Leica EM UC7 ultramicrotome. The ultrathin sections (70 nm) were collected on 100-mesh formvar-coated molybdenum grids. The energy spectrum of the samples was collected at 200 kV on a JEM-2100F transmission electron microscope equipped with a OneView Camera (Gatan Inc., Pleasanton, CA). The preparation of electron microscope slices was completed by the instrument platform of the School of Life Sciences, Peking University. Samples were observed by a field emission high resolution transmission electron microscope (JEM-2100F, Japan) at PKUAIC, Beijing, China. The regions of intercellular space, chloroplast, mitochondrion, nuclei, and intracellular space were visualized under TEM, and then the elemental contents in the selected area were measured by coupling EDS (*46*). The relative content is reflected by calculating the copper content of the same size area, and at least 25 spaces were detected by EDS for each rice line.

### Isolation of the apoplastic wash from rice leaves

Apoplast extraction was performed as previously described (*47*). Four- to 5-week-old rice plants were cut with scissors, and the collected leaves were washed with 0.1% Tween 20 to remove soil particles and then patted dry on paper towels. The rice leaves were then placed in a 100-ml syringe containing 20 ml of VIB buffer [20 mM 2-(*N*-morpholino)ethanesulfonic acid (MES) hydrate, 2 mM CaCl_2_, 0.1 M NaCl, pH adjusted to 6.0]. Vacuum was applied to the leaves for 30 min; excess VIB buffer was removed with a paper towel. Twenty leaves infiltrated with VIB buffer were pushed into a 1-ml syringe that was then placed into a 50-ml conical tube for centrifugation for 10 min at 1000*g* at 4°C. The apoplastic wash was collected from the bottom of the tube and transferred to a new tube. At least 10 rice plants were mixed and used for one biological replicate.

### Protein purification, MST, and EMSAs

The DNA binding domain of SPL9 (SPL9 SBP) was purified as previously described (*18*). The coding sequence of *SPL9 SBP* was amplified by PCR and then inserted into the pGEX4T1 vector (GE Healthcare, Chicago, IL, USA) to induce the production of the glutathione *S*-transferase fusion protein (GST-SPL9 SBP) in Transetta cells (DE3, Transgene Biotech, Beijing, China). The GST fusion proteins were purified with Glutathione Sepharose 4B beads (GE Healthcare); the GST tag was removed by on-column thrombin cleavage. The MST assays were conducted as previously described (*18*). To detect interactions between SPL9 SBP and copper ions or calcium ions, the concentration of SPL9 SBP was held constant, whereas the concentrations of copper or calcium ions were gradient diluted. After a brief incubation for 5 min, the samples were loaded into MST standard glass capillaries. The measurements were performed at 25°C using 20% light-emitting diode power and 20% MST power in an MST machine (NanoTemper Technologies, München, Germany). Data analyses were performed using the NanoTemper Analysis and MO Affinity software provided by the manufacturer. For EMSAs, oligonucleotide probes containing the GTAC motifs in the *miR528* promoter were synthesized and labeled with biotin (Ruibio, Beijing, China). SPL9 SBP was mixed with the biotin-labeled DNA probe and added to gradient-diluted copper or calcium ions. EMSA detection was performed using the Chemiluminescent EMSA Kit (Thermo Fisher Scientific). The probe sequences are listed in table S1.

### AO activity assay and ROS detection

The measurement of AO activity was performed on the basis of the light absorption value of ascorbic acid (AsA), which is oxidized by AO. One hundred milligrams of rice leaves was collected and ground to a powder in liquid nitrogen and then homogenized with 1 ml of 100 mM phosphate-buffered saline pH 6.5 at 4°C for 20 min. The extracts were centrifuged at 15,000*g* for 10 min at 4°C. Ten microliters of the supernatant containing AO was mixed with 80 μl of 100 mM sodium phosphate buffer (pH 5.6) and 10 μl of 2 mM reduced AsA. The absorbance at 265 nm was measured on a Multifunctional Microplate Reader (BioTek). AO activity is normalized to that of NPB.

For ROS detection, H_2_O_2_ was measured using the Amplex Red Hydrogen Peroxide/Peroxidase Assay kit (Invitrogen) following the manufacturer’s directions. 3,3′-Diaminobenzidine and nitroblue tetrazolium staining were performed to test the content of H_2_O_2_ and O_2_^•−^ as described previously (*7*).

### Rice protoplast preparation and transient expression

Rice protoplasts were isolated from 14-day-old rice seedlings as previously described (*54*). The construct pGreenII0800:miR528promoter:luciferase was used as the reporter construct, while the constructs pCAMBIA2300:actin:MYC-SPL9 and pCAMBIA2300 were used as effectors. The appropriate combinations of reporter and effector plasmids were cotransfected into protoplasts. The leaf sheaths were cut into pieces and suspended in enzyme solution [0.6 M mannitol, 10 mM MES, 1.25% (w/v) cellulase R10, 0.3% (w/v) macerozyme R10, 0.1% bovine serum albumin, 1 mM CaCl_2_, 5 mM β-mercaptoethanol, carbenicillin (50 μg/mL) (pH 5.7)] for 4 hours at 28°C to digest the cell wall. The sample was then passed through a 70-μm cell strainer. The flow-through was mixed with an equal volume of W5 solution [154 mM NaCl, 125 mM CaCl_2_, 5 mM KCl, 2 mM MES (pH 5.7)] and centrifuged for 10 min at 150*g* to pellet the protoplasts. The protoplasts were washed in 10 ml of W5 solution twice. The protoplasts were then resuspended in MMG solution [0.6 M mannitol, 15 mM MgCl_2_, 4 mM MES (pH 5.7)]. For transfection, 100 μl of protoplasts, 110 μl 40% polyethylene glycol (PEG) [0.6 M mannitol, 100 mM CaCl_2_, 40% (w/v) PEG 3350], and 20 μg of plasmid DNA were mixed for 20 min in the dark at room temperature. After transfection, the protoplasts were washed and resuspended in W5 solution and incubated at 28°C in the dark overnight. Copper ions were then added to the protoplast suspension. The activities of firefly and *Renilla* luciferases were measured on a GloMax 20/20 Luminometer (Promega). The ratio of firefly to Renilla luciferase activity was calculated to determine transcriptional activity.

### Chloroplast and nuclei extraction

Chloroplasts were isolated from mock-inoculated and RSV-infected rice leaves as previously described (*58*). Rice leaves were homogenized in chloroplast isolation buffer (CIB) [0.5 M sorbitol, 5 mM MgCl_2_, 5 mM EGTA, 5 mM EDTA, 20 mM Hepes/KOH (pH 7.5), and 10 mM NaHCO_3_]. The extract was filtered through a 70-μm cell strainer. The flow-through was centrifuged for 3 min at 200*g*. Then, the supernatant was centrifuged for 10 min at 1000*g* to pellet the chloroplast. The chloroplasts were then resuspended in CIB solution and added to the Percoll solution (40%) containing CIB and centrifuged at 7000*g* for 10 min. The intact chloroplasts were collected at the bottom of the Percoll solution.

Nuclei isolation was performed as described previously (*59*). Mock-inoculated and RSV-infected rice leaves (about 1 g) were harvested, homogenized into powder in liquid nitrogen, and suspended in lysis buffer [0.25 M sucrose, 25 mM tris-HCl (pH 7.5), 10 mM MgCl_2_, 0.5% Triton X-100, 0.5 mM phenylmethylsulfonyl fluoride,1 mM dithiothreitol, and Roche protease inhibitor cocktail] (2 ml/g). The extract was then filtered through double layers of Miracloth twice. The flow-through was centrifuged at 3000*g* for 20 min at 4°C to pellet the nuclei. The pellet was then washed four times with lysis buffer to collect the nuclei.

### Determination of copper content in rice leaf by XRF

Rice leaves from mock-inoculated and RSV-infected rice plants were cut to about 3 cm long and fixed on the detection module (as shown in fig. S4A). Then, the leaves were scanned according to the excitation peak position of copper ions, and the height of the peak reflected the relative content of copper ions (as shown in fig. S4B). The relative copper content is reflected by calculating the copper content of the same size area; this detection was completed by x-ray fluorescence spectrometer (Zetium, Netherlands) at the Analytical Instrumentation Center of Peking University (PKUAIC, Beijing, China). At least six leaves were detected by XRF for each rice line.

### Phylogenetic analysis

The sequences of SPL and COPT family protein from rice and Arabidopsis were obtained from UniProt (www.uniprot.org/). An unrooted, maximum likelihood tree was constructed using MEGA 7.

### Quantification and statistical analysis

Statistical significance of results of copper content analysis by ICP-OES and TEM-EDS, RT-qPCR, and the luciferase reporter system is determined by Student’s *t* test (∗*P* ≤ 0.05, ∗∗*P* ≤ 0.01, and ∗∗∗*P* ≤ 0.001) or Tukey’s test (different letters indicate significant difference, *P* ≤ 0.05). All values for copper content analysis by ICP-OES and TEM-EDS, RT-qPCR analysis, the luciferase reporter system, RSV disease symptom classification statistics, and MST assays are presented by means ± SD. The number (*n*) of biological replicates is indicated in the legends. For immunoblot quantification analysis, band intensities are quantified by ImageJ software (*60*).

## Supplementary Materials

### This PDF file includes:

Figs. S1 to S11 Tables S1 to S4 References

## Notes

View/request a protocol for this paper from *Bio-protocol*.
