# Supplementary material for: The key micronutrient copper orchestrates broad-spectrum virus resistance in rice
Source: Sci Adv. 2022 Jul 1;8(26):eabm0660. doi: 10.1126/sciadv.abm0660 (PMC10883364; doi:10.1126/sciadv.abm0660)
Supplement: Supplementary file 1 — Figs. S1 to S11 Tables S1 to S4 References [file sciadv.abm0660_sm.pdf]

Science Advances
AAAS

Supplementary Materials for

The key micronutrient copper orchestrates broad-spectrum virus resistance in rice

Shengze Yao *et al.*

Corresponding author: Yi Li, liyi@pku.edu.cn

Sci. Adv. **8**, eabm0660 (2022)
DOI: 10.1126/sciadv.abm0660

This PDF file includes:

Figs. S1 to S11
Tables S1 to S4
References

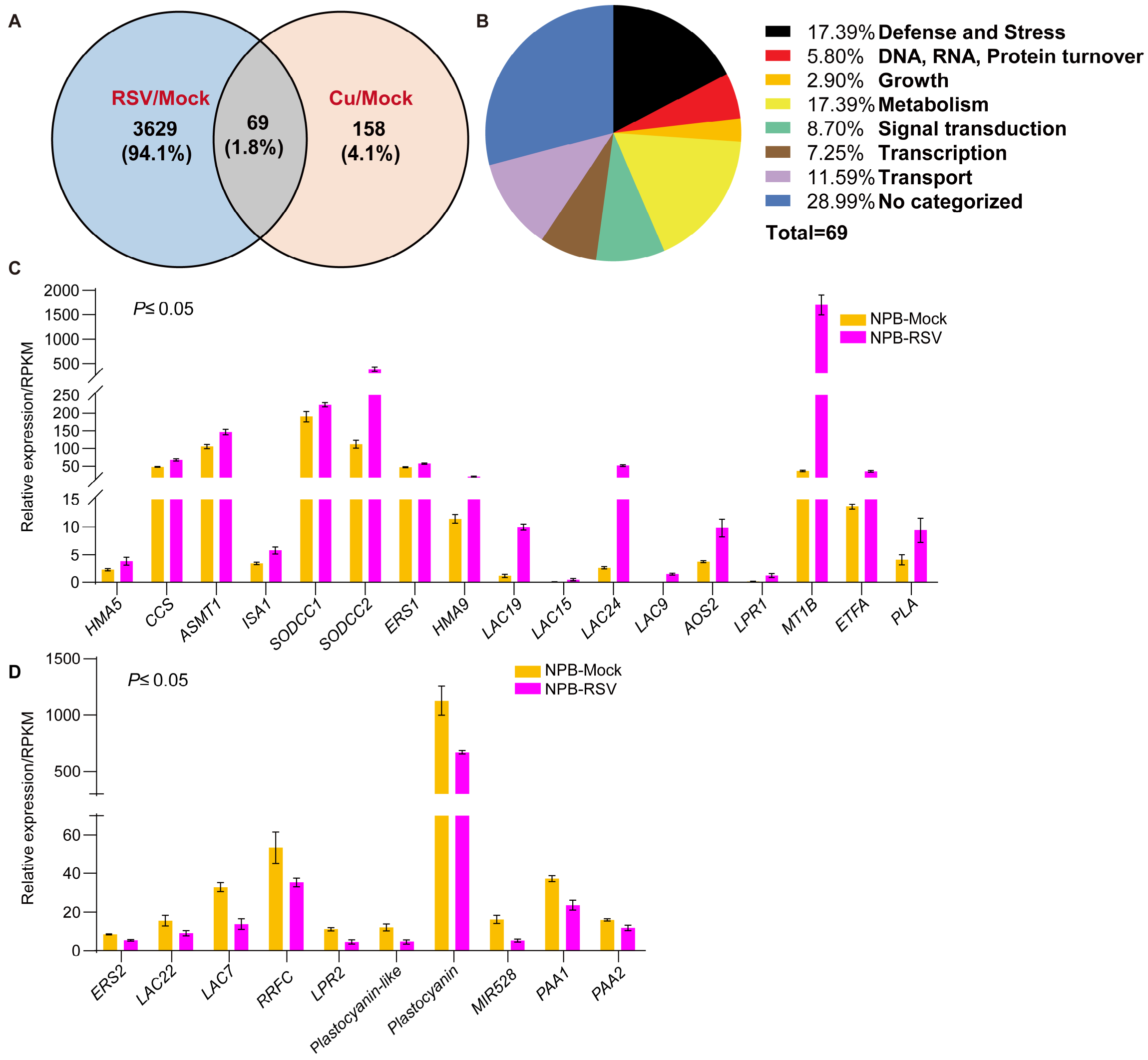

fig. S1 Copper may be a key regulator during viral infection. (**A**) Venn diagram showing the overlap between DEGs (differentially expressed genes) in RSV-infected versus mock-inoculated (*17*) and copper-treated versus mock-inoculated NPB (*44*). A total of 69 genes significantly responded to both treatments. (**B**) Analysis of the physiological processes associated with the 69 DEGs. (**C, D**) Genes encoding copper transporters and copper-binding proteins are significantly induced (C) or suppressed (D) by RSV infection ($P \leq 0.05$).

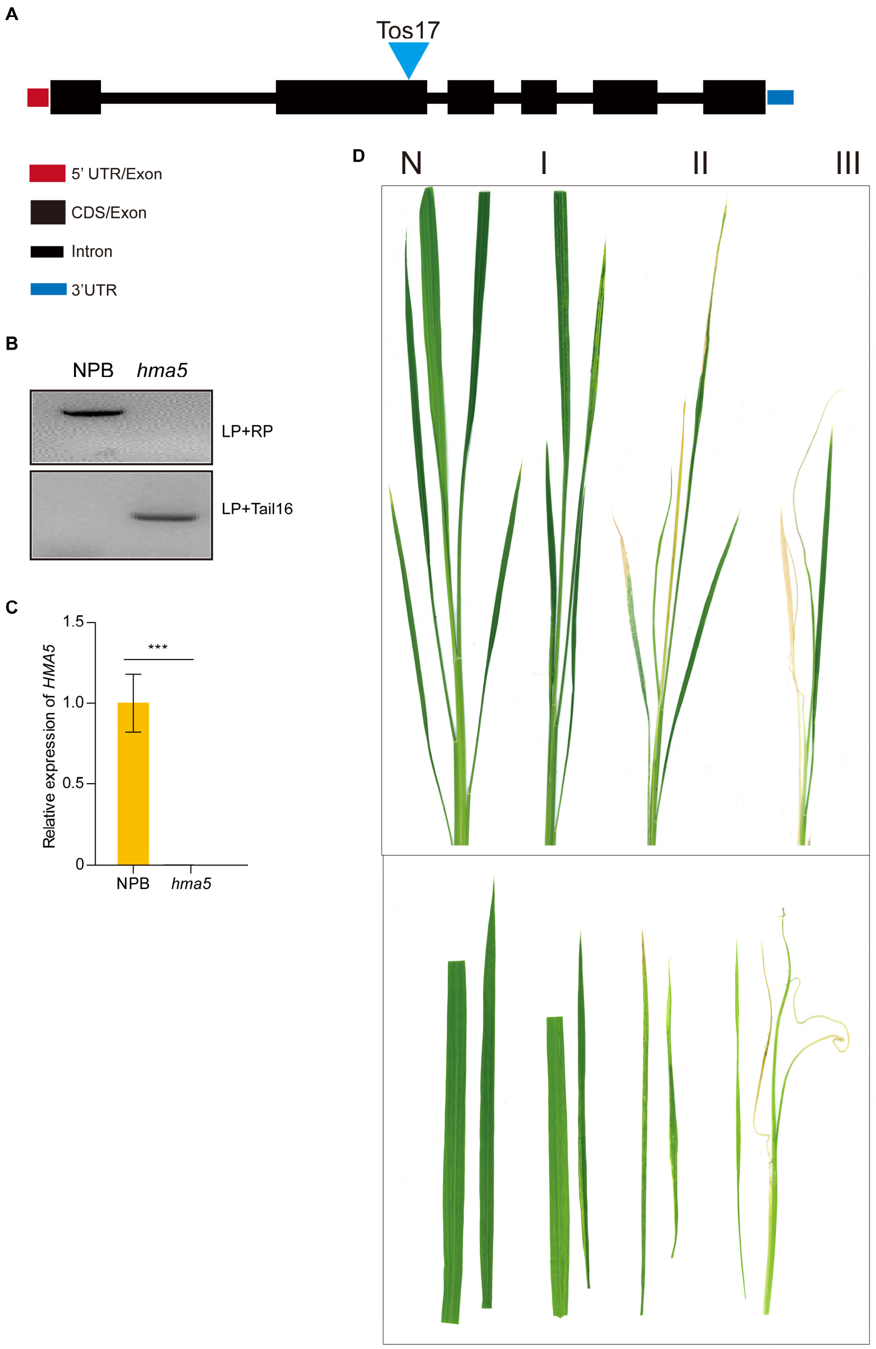

fig. S2 Identification of the *hma5* mutant and different disease symptom grades upon RSV infection. (**A**) Schematic diagram of the T-DNA insertion into the *HMA5* locus in the *hma5* mutant. (**B**) PCR verification of the *hma5* mutant. (**C**) RT-qPCR analysis of *HMA5* transcript levels in NPB and *hma5* plants. (**D**) Photographs of rice plants with different grades of disease symptoms at 4 wpi. N, no noticeable disease symptoms; I, mild symptoms on leaves; II, typical yellow-green stripes on leaves; III, curled or dead new leaf.

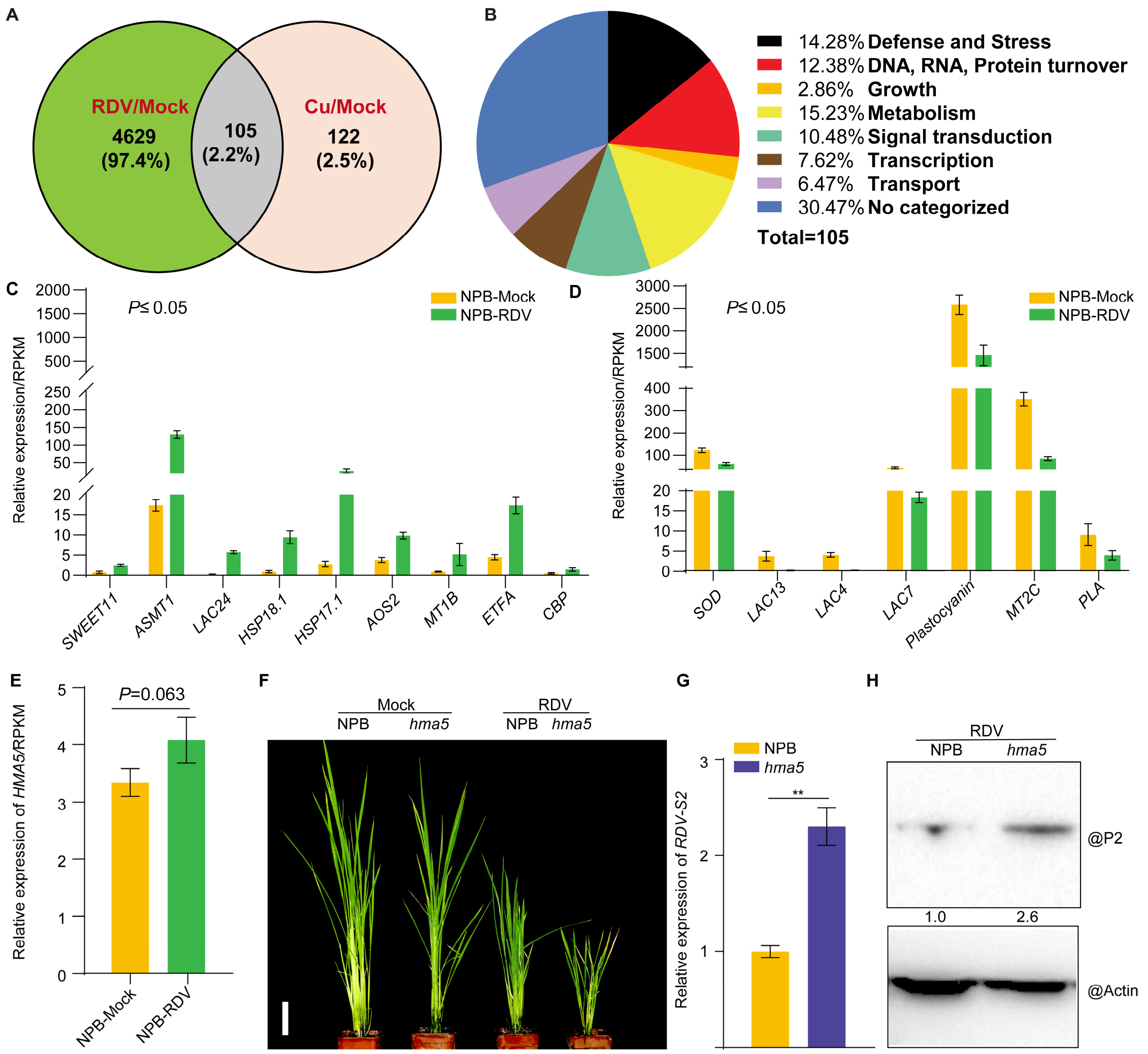

fig. S3 Copper regulates rice resistance to RDV. (**A**) Venn diagram showing the overlap between DEGs in RDV-infected versus mock-inoculated (*45*) and copper-treated versus mock-inoculated NPB (*44*). A total of 105 genes significantly responded to both treatments. (**B**) Analysis of the physiological processes associated with the 105 DEGs. (**C** and **D**) Genes encoding copper transporters and copper-binding proteins are significantly induced (C) or suppressed (D) by RDV infection ($P \leq 0.05$). (**E**) *HMA5* expression detected by RNA-seq in mock-inoculated and RDV-infected NPB plants. (**F**) Symptoms of mock-inoculated or RDV-infected NPB and *hma5* at 4 wpi. Scale bar, 10 cm. (**G**) RT-qPCR analysis of *RDV-S2* transcript levels in RDV-infected NPB and *hma5*. (**H**) RDV-P2 abundance in RDV-infected NPB and *hma5* by immunoblotting. Actin was used as loading control. Data are shown as mean ± SD (n = three biological repeats). Asterisks mark significant differences according to Student's *t*-test: $**P \leq 0.01$.

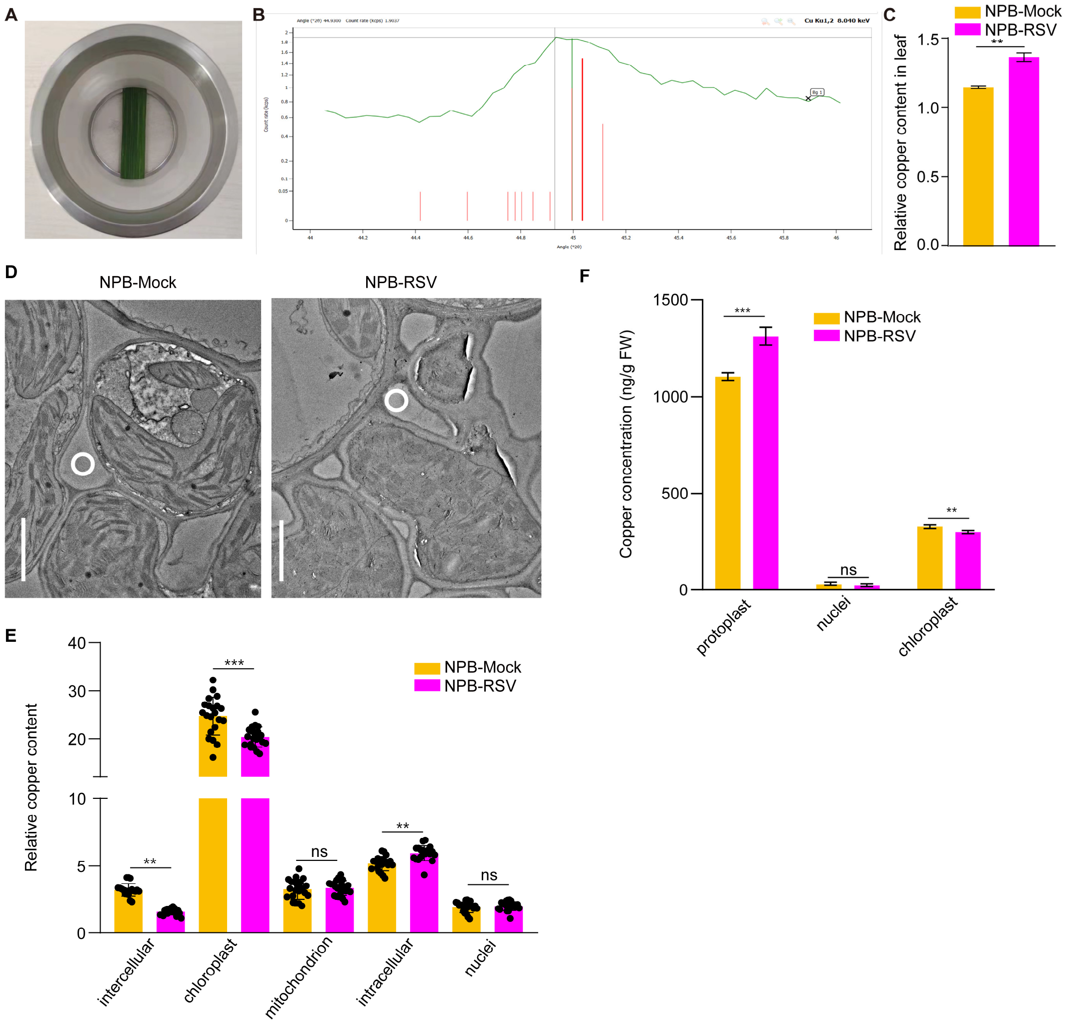

fig. S4 Copper location was changed during viral infection. (**A**) Images of healthy NPB leaf used for copper detection by XRF. (**B**) Scanning peaks of copper ions shown in (A). (**C**) Copper content in leaves of mock-inoculated or RSV-infected NPB, as measured by XRF. (**D**)Transmission electron microscopy (TEM) images of healthy and RSV-infected NPB leaf cells. The white circle indicates intercellular space between adjacent cells in which copper was detected. Scale bar, 2 μm. (**E**) Relative copper content of the intercellular space, chloroplast, mitochondrion, intracellular space, and nuclei detected by EDS in mock-inoculated or RSV-infected NPB leaf cells. Each dot represents one detection space. (**F**) Copper concentration in the protoplast, nuclei, and chloroplast of mock-inoculated or RSV-infected NPB, as measured by ICP-OES. FW, fresh weight. Data are shown as means ± SD (n = three biological repeats). Asterisks mark significant differences according to Student's *t*-test: ns, no significant difference; * *$P \leq 0.01$; *** $P \leq 0.001$.

A

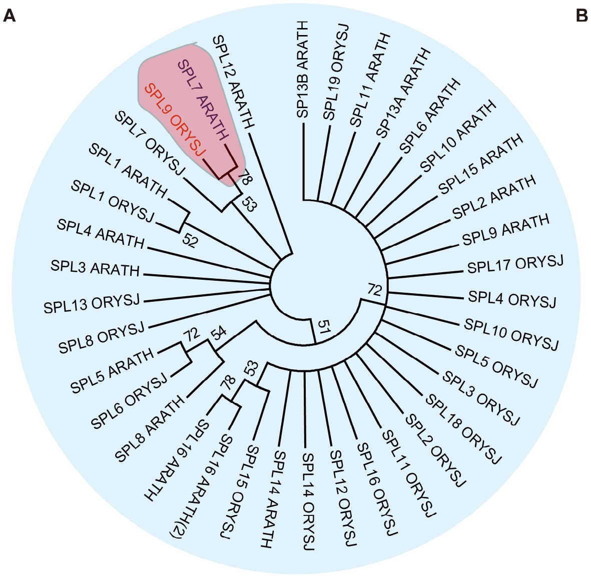

B

Major Target genes of Os-miR528		
**Target_Accession**	**Target_Description**	**Reference**
LOC_Os06g06050	OsFBL27 - F-box domain and LRR containing protein	(*61*)
LOC_Os08g04310	OsUCL23, plastocyanin-like domain containing protein	(*62*)
LOC_Os06g37150	L-ascorbate oxidase (AO)	(*7*)
LOC_Os08g42640	RFI2, 3HC4 type domain containing protein	(*54*)

fig. S5 The SPL9-miR528-AO pathway is associated with copper regulation. (**A**) Phylogenetic analysis of all SPL proteins in rice and Arabidopsis. An unrooted tree was constructed by aligning all SPL protein sequences in rice and Arabidopsis. The maximum likelihood method was used to generate the phylogenetic tree. Rice SPL9 and Arabidopsis SPL7 are highlighted. (**B**) Target genes of miR528 that were experimentally validated in rice.

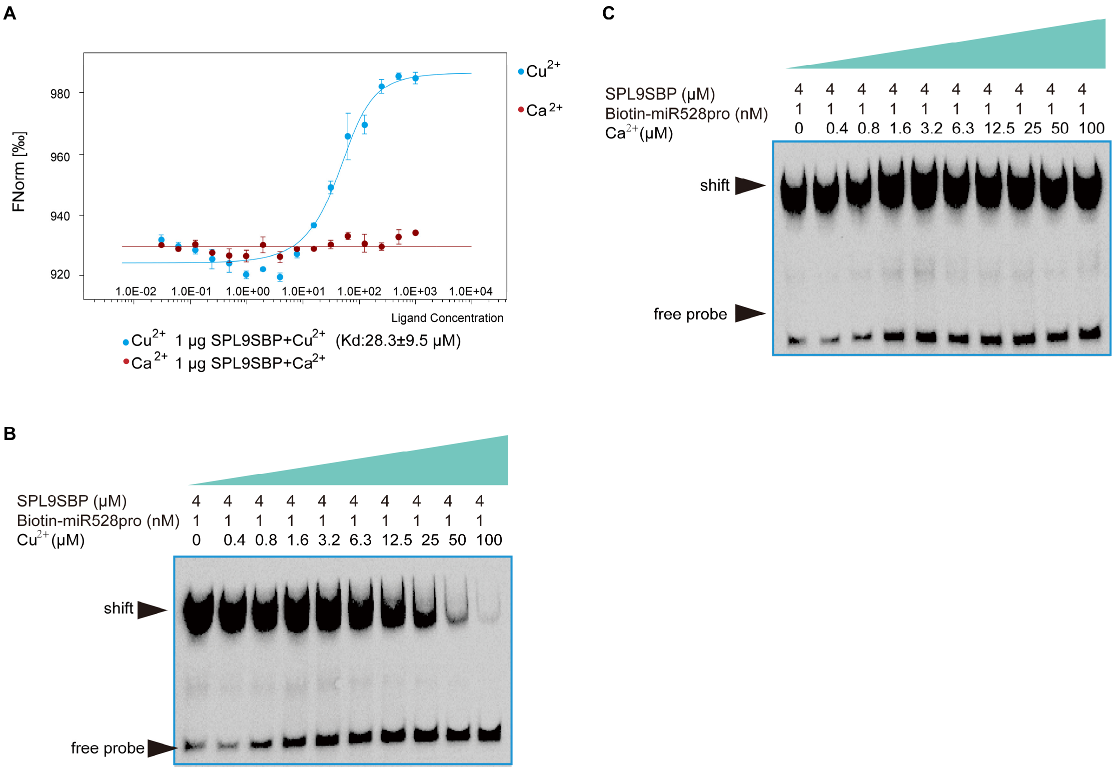

fig. S6 Copper regulates SPL9-mediated transcription of *miR528*. (**A**) SPL9 binds to copper. *K*d values for SPL9-copper binding were determined by microscale thermophoresis (MST). (**B, C**) The biotinylated probe containing the GTAC motif sequence of miR528 promoter was incubated with recombinant SPL9 DNA-binding domain (SPL9 SBP), together with increasing concentrations of copper (B) or calcium (C).

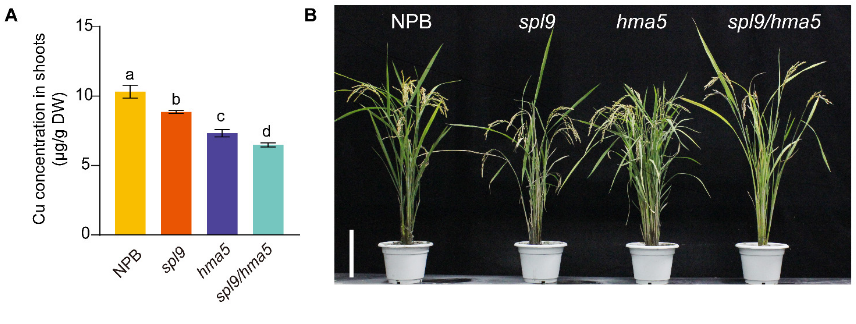

fig. S7 HMA5 and SPL9 positively regulates copper accumulation in rice. (**A**) Copper concentration in shoot of NPB, *spl9*, *hma5*, and *spl9/hma5*, as measured by ICP-OES. DW, dry weight. (B) Phenotypes of NPB, *spl9*, *hma5*, and *spl9/hma5* at reproductive growth stage. Scale bar, 25 cm. Data are shown as means ± SD (n = three biological repeats). Tukey's test was performed for multiple comparisons; different letters indicate significant differences between the compared pairs ($P \leq 0.05$).

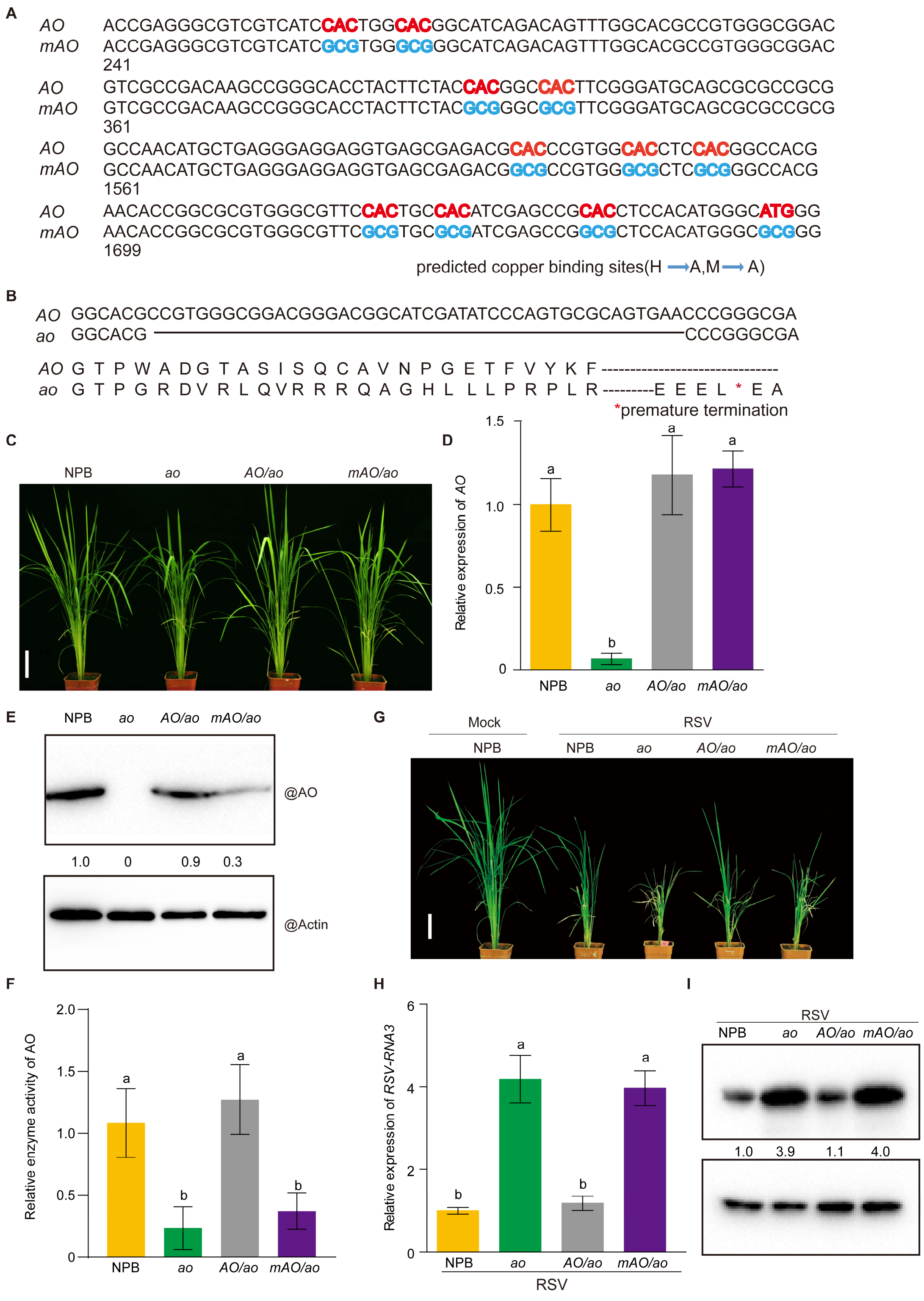

fig. S8 The antiviral function of AO relies on copper. (**A**) Schematic diagram of the mutation of the copper ion binding sites in AO. (**B**) The generation of *ao* mutant lines using CRISPR/Cas9-mediated genome editing. The DNA sequences of the *ao* mutant lines are shown. The mutation, a 44-bp deletion in *ao*, caused a premature termination of translation. (**C**) Phenotypic comparison of NPB, *ao*, *AO/ao*, and *mAO/ao* plants at 6 weeks after germination. Scale bar, 10 cm. (**D**) RT-qPCR analysis of the relative transcript levels of *AO* in NPB, *ao*, *AO/ao*, and *mAO/ao* plants at 6 weeks after

germination. (**E**) Detection of AO protein abundance in NPB, *ao*, *AO/ao*, and *mAO/ao* plants at 6 weeks after germination by immunoblotting. Actin was used as loading control. (**F**) Relative AO activity in NPB, *ao*, *AO/ao*, and *mAO/ao* rice plants. (**G**) Symptoms of mock-inoculated NPB and RSV-infected NPB, *ao*, *AO/ao*, and *mAO/ao* lines at 4 wpi (weeks post inoculation). Scale bar, 10 cm. (**H**) RT-qPCR analysis of *RSV-RNA3* transcript levels in RSV-infected NPB, *ao*, *AO/ao*, and *mAO/ao* lines. (**I**) Detection of RSV-CP protein in RSV-infected NPB, *ao*, *AO/ao*, and *mAO/ao* lines by immunoblotting. Actin was used as loading control. The average values (±SD) from three biological repeats are shown. Tukey's test was performed for multiple comparisons; different letters indicate significant differences between the compared pairs ($P \leq 0.05$).

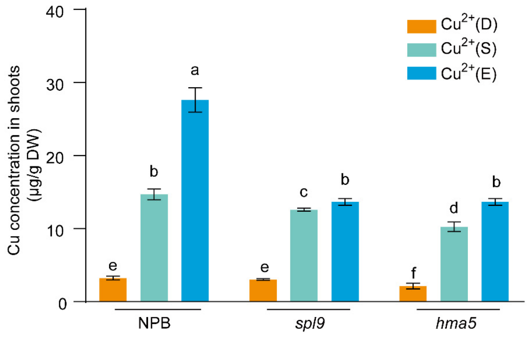

fig. S9 Copper accumulation level detection in rice plants treated by copper solutions. Copper concentration in shoot of NPB, *spl9*, and *hma5* plants treated by the indicated exogenous copper concentrations as measured by ICP-OES. DW, dry weight. Data are shown as means ± SD (n = three biological repeats). Tukey's test was performed for multiple comparisons; different letters indicate significant differences between the compared pairs ($P \leq 0.05$).

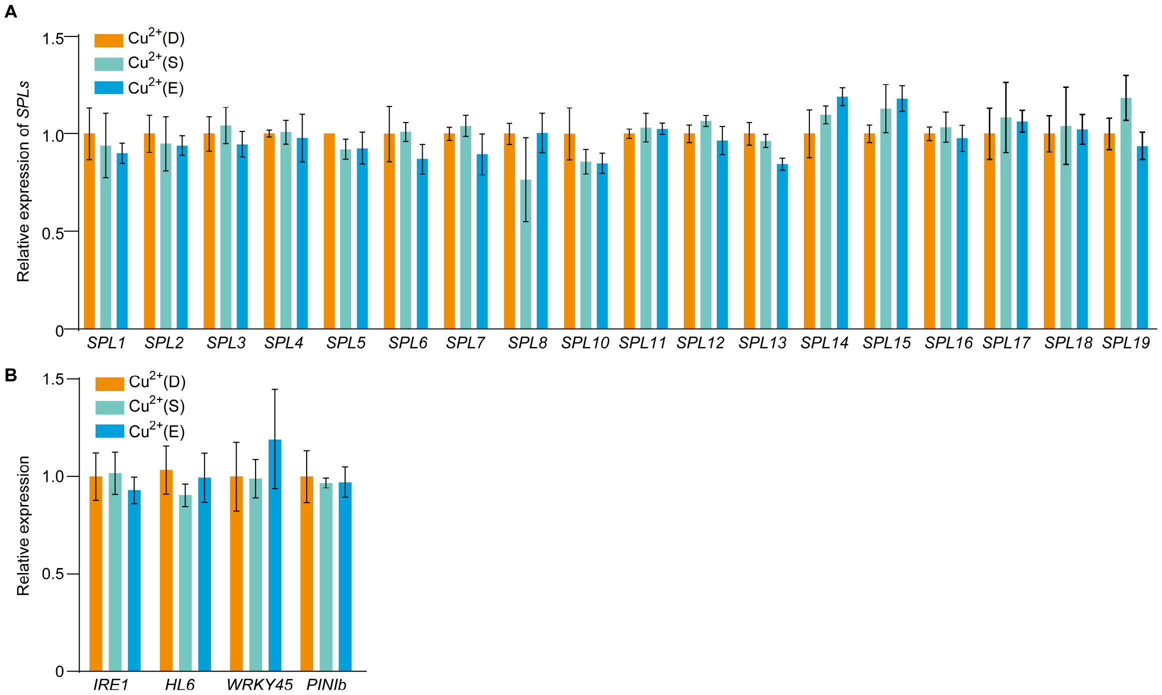

fig. S10 The regulation of SPL9 by copper is specific. (**A**) RT-qPCR analysis of *SPL* transcript levels in NPB plants exposed to the indicated exogenous copper concentrations at 6 weeks after germination. (**B**) RT-qPCR analysis of *IRE1* (transcriptionally regulated by SPL6), *HL6* (transcriptionally regulated by SPL10), *WRKY45* (transcriptionally regulated by SPL14), and *PINIb* (transcriptionally regulated by SPL17) transcript levels in NPB plants exposed to the indicated exogenous copper concentrations. The average values (±SD) from three biological repeats are shown. There is no significant difference in these genes across treatments.

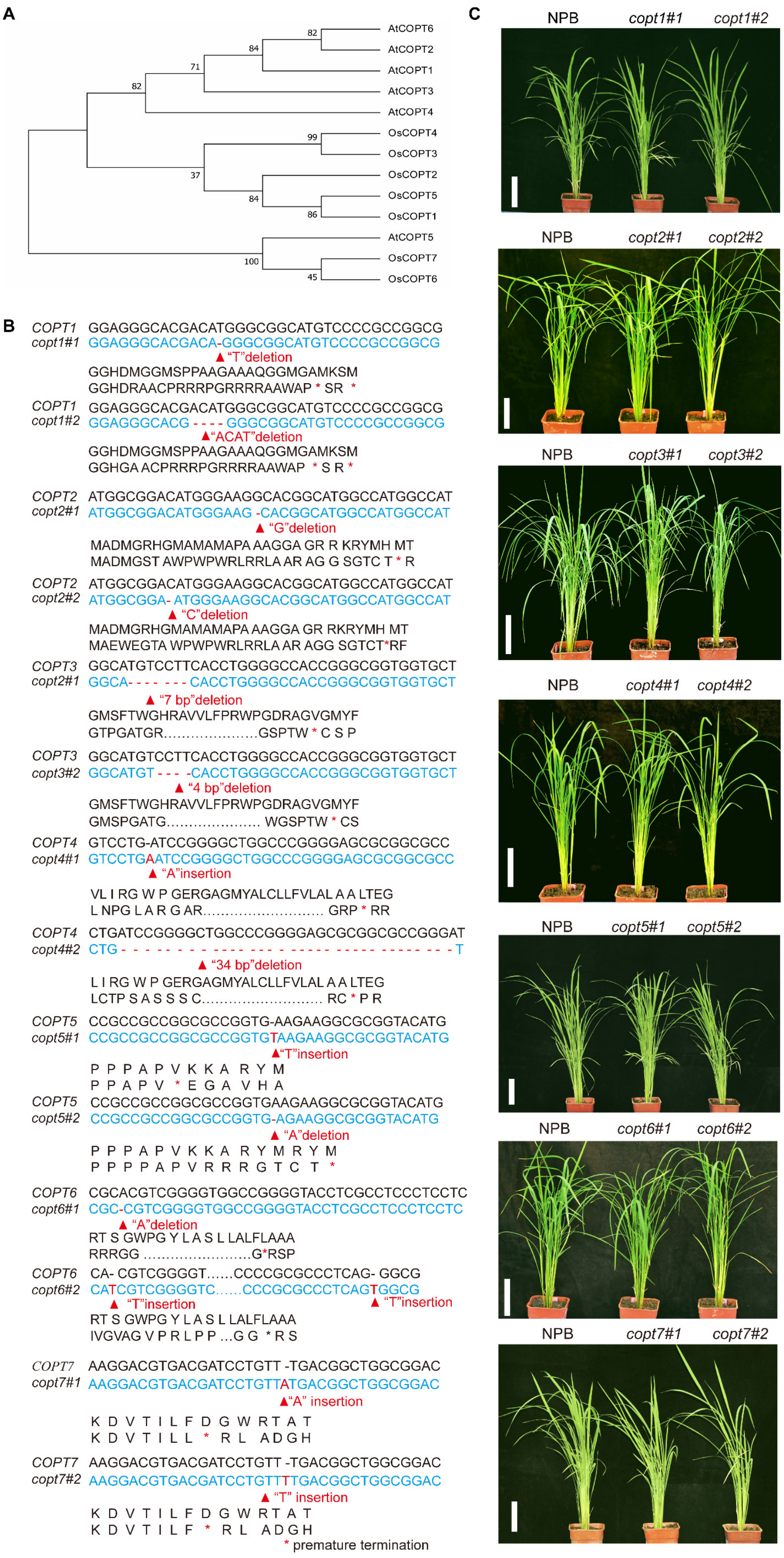

fig. S11 Generation of mutants in each member of the *COPT* family. (**A**) Phylogenetic analysis of the seven rice COPT and six Arabidopsis COPT proteins. An unrooted tree was constructed by aligning all COPT protein sequences. The maximum likelihood method was used to generate the phylogenetic tree. (**B**) Generation of single *copt* mutant lines using CRISPR/Cas9-mediated genome editing. The DNA sequences of the *copt*

mutant lines are shown. The mutations in *copt* lines introduced a premature termination of translation. (**C**) Representative photographs of healthy NPB and *copt* lines. Scale bar, 10 cm.

Table S1. Primers used in this study.

Primer	Sequence	Purpose
2300-SPL9-F	TCTACATCCCGGGGATCCTCTAGAGAT GGACGCCCCCGGCGGCG	Cloning for luciferase assays
2300-SPL9-R	ATTAAAGCAGGGCATGCCTGCAGCTAT GATGAGTAGTTCCTAGAC	
0800-528pro-F	CTATAGGGCGAATTGGGTACCCTTTTA TAAATACATGACAGC	
0800-528pro-R	AGTGGATCCCCCGGGCTGCAGATTGGT GAAGGGTGGCATATTT	
EF1α-F	ACATTGCCGTCAAGTTTGCTG	RT-qPCR
EF1α-R	AACAGCCACCGTTTGCCTC	
SPL9-F	AGCAGATGGTAGTGGTGATG	
SPL9-R	CAGAAGCAGCGAAAGTCA	
AO-F	CGAGAACGTGGAGACCTGCGTCGA	
AO-R	CCACCACCGTCATCTTGTGCCCTTG	
pre-miR528-F	CAGAGGAGCAGGAGATTCA	
pre-miR528-F	AAACTTCCACAGAACAGCCT	
RSV-RNA1-F	GCACCCAATAGGTATCTCCTTGAT	
RSV-RNA1-R	CAAATGACCCTACTAGATGGACGA	
RSV-RNA3-F	TATATGGGCACCAACAAGCCAGCC	
RSV-RNA3-R	TATGACTTAGGGAGTGAGTTGTGCAGT	
COPT1-F	CATGGGCGCCATGAAGTC	
COPT1-R	GTGAAGAGCACCTCCGAGTTCT	
COPT2-F	CGGAGATCCTCTTCACCGG	
COPT2-R	CTGCCGAGGGAGGAGGAC	
COPT3-F	GGATGGGGCTCGCCTACC	
COPT3-R	GGTTTCGGCTCTGACGATGG	
COPT4-F	CGCCGCAGCACAAGATGGC	
COPT4-R	GCAGAGGGCGTACATCCCG	
COPT5-F	GCTGTCTCGCTCGTCATGGT	
COPT5-R	CGCACACACAAAACATCAACAA	
COPT6-F	GGTACCTCGCCTCCCTCC	
COPT6-R	GGCACGACGGGACCGGCG	
COPT7-F	GCCTAGGGTTTGGCTTTGC	
COPT7-R	ACAAGATCGGGAAACCAAACA	

Primer	Sequence	Purpose
HMA5-F	AAGGTGGAGAGTATAATGGTGAC	RT-qPCR
HMA5-R	CCTTCCGGCCGACTGAAGTTC	
SPL1-F	ATGTCGAGTGGGCTCAAGAAG	
SPL1-R	TCACTTGGGGCCTGAACGC	
SPL2-F	ATGGATTGGGACGCCAAG	
SPL2-R	CTA CCA CGA TGA GAA AGG AAG	
SPL3-F	ATG GGT TCT TTT GGG ATG GAC	
SPL3-R	TCA GTT CAT CTG ATC ATA GTG	
SPL4-F	ATGGATTGGATGCCTCCTCC	
SPL4-R	TTA ATG AAA TGA CAT GCA GC	
SPL5-F	ATG GCG GTG CCA GCG GCG G	
SPL5-R	CTA GAT GAA ATC CAC CTC GA	
SPL6-F	ATGGAGGCTGCCCGGGTCGG	
SPL6-R	TCA CAT TGG TCC ACG TTC TAA	
SPL7-F	ATGGAAGGAAACGGCTGCGG	
SPL7-R	TCA GAC CAC GCG GGC GCC CTC	
SPL8-F	ATGATGAACGTTCCATCCGC	
SPL8-R	CTA GTG ATC GAA GTC GAG ATC	
SPL10-F	CCAGCAATGCAGCAGGTTTC	
SPL10-R	TTCCGGCAGCTCCTCTTG	
SPL11-F	ATGGAGTGCAACCCCGTCTC	
SPL11-R	TCA ATG TAT CTG GTT CAG AC	
SPL12-F	ATGGCTTCTTTTGGGATGAAC	
SPL12-R	TCA GTG CAG ATG GCC ATA GCC	
SPL13-F	ATGGACCGCAAGGACAAGGC	
SPL13-R	TTA TCT GAT CTG GAA CGG CGG	
SPL14-F	ATGGAGATGGCCAGTGGAGG	
SPL14-R	CTA CAG AGA CCA ATC CAT CG	
SPL15-F	ATGCAGAGGGAAGTGGGGCCG	
SPL15-R	TTA TAT CGT ACC AAA ATC CA	
SPL16-F	ATGGAGTGGGATCTCAAGATG	
SPL16-R	CTA CTG CCA TGA GAA CGG CAG	
SPL17-F	CCACAAGGTGTGCTACATGC	
SPL17-R	GGCCATAGCGAGAAGAAAGA	

Primer	Sequence	Purpose
SPL18-F	ATGGATTGGGATCTCAAGATG	RT-qPCR
SPL18-R	CTACTGCCACGAGAATGGGAG	
SPL19-F	ATGGAGTGGGCGGCGGCGG	
SPL19-R	CTACACCTGCCAAGAGAATG	
HL6-F	CCTGCTTCTGCATTTGACACCTA	
HL6R	CCGCTGTGGCTGCTAGTC	
PINIb-F	TGCACCCTAGCATTCTCAGCA	
PINIb-R	CCCTCCTCCCAAATTCTACTT	
IRE1-F	TGCCCTGCTTGCAGACATGG	
IRE1-R	GACCCAAGTAGCTCCTTGAGATC	
WRKY45-F	CGGGTAAAACGATCGAAAGA	
WRKY45-R	TTTCGAAAGCGGAAGAACAG	
Lablled-miR528	Biotin-CTCCTCTGCATGCCCCTTCCA	miRNA northern blot
Lablled-U6	Biotin-TTCCCGATCGGTCACCCATCCCAAAATTGCT	
Tail16	GGTGAAAAGGACAGTGGAGCA	*hma5* identification
LP	CTCCTCACATAATTACATGGCAAC	
RP	TCAACGGCCCAGTTTTGGGCT	

Table S2. sgRNA used for mutant construction by CRISPR/Cas9 in this study.

Mutant	sgRNA Sequence
spl9	TTGAATGATACACGTCCTGT
spl9/hma5	CCGTGGGGGGACGATTCGATTGG
copt1	ACCATGTGGCCCGGCACGAGGGG
copt2	ATGGCGGACATGGGAAGGCACGG
copt3	CCGACGGGCATGTCCTTCACCTGG
copt4	ACCGCGCGGTGGTCCTGATCCGG
copt5	ATGTACCGCGCCTTCTTCACCGG
copt6	TTCGACGGCTGGCGCACGTCGGG
copt7	GATCCGCGTCAAGCTCCTCGCGG
ao	TCCCAGTGCGCAGTGAACCC

Table S3. Gene information in this study.

Gene	Locus number
SPL1	LOC_Os01g18850
SPL2	LOC_Os01g69830
SPL3	LOC_Os02g04680
SPL4	LOC_Os02g07780
SPL5	LOC_Os02g08070
SPL6	LOC_Os03g61760
SPL7	LOC_Os04g46580
SPL8	LOC_Os04g56170
SPL9	LOC_Os05g33810
SPL10	LOC_Os06g44860
SPL11	LOC_Os06g45310
SPL12	LOC_Os06g49010
SPL13	LOC_Os07g32170
SPL14	LOC_Os08g39890
SPL15	LOC_Os08g40260
SPL16	LOC_Os08g41940
SPL17	LOC_Os09g31438
SPL18	LOC_Os09g32944
SPL19	LOC_Os11g30370
MIR528	LOC_Os03g03724
AO	LOC_Os06g37150
COPT1	LOC_Os01g56420
COPT2	LOC_Os01g56430
COPT3	LOC_Os03g25470
COPT4	LOC_Os04g33900
COPT5	LOC_Os05g35050
COPT6	LOC_Os08g35490
COPT7	LOC_Os09g26900
HMA5	LOC_Os04g46940

Table S4. Non-preference test for copper on rice plants with indicated.

Varieties	Non-preference[*1]
Copper-deficiency (NPB)	1.83 [a*2]
Copper-sufficient (NPB)	1.78 [a]
Copper-excess (NPB)	1.81 [a]
NPB	1.75 [a]
spl9	1.80 [a]
hma5	1.83 [a]
spl9/hma5	1.79 [a]

*1, Non-preference was indicated by the number of small brown planthopper settled on the individual plant.

*2, "a" means there is no significant difference (P value> 0.05) between these data.

REFERENCES AND NOTES

1. S. Muthayya, J. D. Sugimoto, S. Montgomery, G. F. Maberly, An overview of global rice production, supply, trade, and consumption. *Ann. N. Y. Acad. Sci.* **1324**, 7–14 (2014).

2. Y. Xu, S. Fu, X. Tao, X. Zhou, Rice stripe virus: Exploring molecular weapons in the arsenal of a negative-sense RNA virus. *Annu. Rev. Phytopathol.* **59**, 351–371 (2021).

3. X. Cao, P. Zhou, X. Zhang, S. Zhu, X. Zhong, Q. Xiao, B. Ding, Y. Li, Identification of an RNA silencing suppressor from a plant double-stranded RNA virus. *J. Virol.* **79**, 13018–13027 (2005).

4. S. Zhu, F. Gao, X. Cao, M. Chen, G. Ye, C. Wei, Y. Li, The rice dwarf virus P2 protein interacts with ent-kaurene oxidases in vivo, leading to reduced biosynthesis of gibberellins and rice dwarf symptoms. *Plant Physiol.* **139**, 1935–1945 (2005).

5. J. Ye, L. Zhang, X. Zhang, X. Wu, R. Fang, Plant defense networks against insect-borne pathogens. *Trends Plant Sci.* **26**, 272–287 (2021).

6. J. Wu, Z. Yang, Y. Wang, L. Zheng, R. Ye, Y. Ji, S. Zhao, S. Ji, R. Liu, L. Xu, H. Zheng, Y. Zhou, X. Zhang, X. Cao, L. Xie, Z. Wu, Y. Qi, Y. Li, Viral-inducible Argonaute18 confers broad-spectrum virus resistance in rice by sequestering a host microRNA. *eLife* **4**, (2015).

7. J. Wu, R. Yang, Z. Yang, S. Yao, S. Zhao, Y. Wang, P. Li, X. Song, L. Jin, T. Zhou, Y. Lan, L. Xie, X. Zhou, C. Chu, Y. Qi, X. Cao, Y. Li, ROS accumulation and antiviral defence control by microRNA528 in rice. *Nat. Plants* **3**, 16203 (2017).

8. D. C. Baulcombe, How virus resistance provided a mechanistic foundation for RNA silencing. *Plant Cell* **31**, 1395–1396 (2019).

9. Z. Guo, Y. Li, S. W. Ding, Small RNA-based antimicrobial immunity. *Nat. Rev. Immunol.* **19**, 31–44 (2019).

10. J. D. G. Jones, J. L. Dangl, The plant immune system. *Nature* **444**, 323–329 (2006).

11. J. M. Zhou, Y. Zhang, Plant immunity: Danger perception and signaling. *Cell* **181**, 978–989 (2020).

12. A. Carbonell, J. C. Carrington, Antiviral roles of plant ARGONAUTES. *Curr. Opin. Plant Biol.* **27**, 111–117 (2015).

13. J. Hu, J. Huang, H. Xu, Y. Wang, C. Li, P. Wen, X. You, X. Zhang, G. Pan, Q. Li, H. Zhang, J. He, H. Wu, L. Jiang, H. Wang, Y. Liu, J. Wan, Rice stripe virus suppresses jasmonic acid-mediated resistance by hijacking brassinosteroid signaling pathway in rice. *PLOS Pathog.* **16**, e1008801 (2020).

14. Z. Ma, X. Zhang, Actions of plant Argonautes: Predictable or unpredictable? *Curr. Opin. Plant Biol.* **45**, 59–67 (2018).

15. F. Qu, X. Ye, T. J. Morris, Arabidopsis DRB4, AGO1, AGO7, and RDR6 participate in a DCL4-initiated antiviral RNA silencing pathway negatively regulated by DCL1. *Proc. Natl. Acad. Sci. U.S.A.* **105**, 14732–14737 (2008).

16. S. Zhao, Y. Li, Current understanding of the interplays between host hormones and plant viral infections. *PLOS Pathog.* **17**, e1009242 (2021).

17. Z. Yang, Y. Huang, J. Yang, S. Yao, K. Zhao, D. Wang, Q. Qin, Z. Bian, Y. Li, Y. Lan, T. Zhou, H. Wang, C. Liu, W. Wang, Y. Qi, Z. Xu, Y. Li, Jasmonate signaling enhances RNA silencing and antiviral defense in rice. *Cell Host Microbe* **28**, 89–103.e8 (2020).

18. S. Yao, Z. Yang, R. Yang, Y. Huang, G. Guo, X. Kong, Y. Lan, T. Zhou, H. Wang, W. Wang, X. Cao, J. Wu, Y. Li, Transcriptional regulation of miR528 by OsSPL9 orchestrates antiviral response in rice. *Mol. Plant* **12**, 1114–1122 (2019).

19. H. Zhang, L. Li, Y. He, Q. Qin, C. Chen, Z. Wei, X. Tan, K. Xie, R. Zhang, G. Hong, J. Li, J. Li, C. Yan, F. Yan, Y. Li, J. Chen, Z. Sun, Distinct modes of manipulation of rice auxin response factor OsARF17 by different plant RNA viruses for infection. *Proc. Natl. Acad. Sci. U.S.A.* **117**, 9112–9121 (2020).

20. Q. Qin, G. Li, L. Jin, Y. Huang, Y. Wang, C. Wei, Z. Xu, Z. Yang, H. Wang, Y. Li, Auxin response factors (ARFs) differentially regulate rice antiviral immune response against rice dwarf virus. *PLOS Pathog.* **16**, e1009118 (2020).

21. L. Jin, Q. Qin, Y. Wang, Y. Pu, L. Liu, X. Wen, S. Ji, J. Wu, C. Wei, B. Ding, Y. Li, Rice dwarf virus P2 protein hijacks auxin signaling by directly targeting the rice OsIAA10 protein, enhancing viral infection and disease development. *PLOS Pathog.* **12**, e1005847 (2016).

22. V. Kumar, S. Pandita, G. P. Singh Sidhu, A. Sharma, K. Khanna, P. Kaur, A. S. Bali, R. Setia, Copper bioavailability, uptake, toxicity and tolerance in plants: A comprehensive review. *Chemosphere* **262**, 127810 (2021).

23. G. Aguirre, M. Pilon, Copper delivery to chloroplast proteins and its regulation. *Front. Plant Sci.* **6**, 1250 (2016).

24. Z. Shabbir, A. Sardar, A. Shabbir, G. Abbas, S. Shamshad, S. Khalid, Natasha, G. Murtaza, C. Dumat, M. Shahid, Copper uptake, essentiality, toxicity, detoxification and risk assessment in soil-plant environment. *Chemosphere* **259**, 127436 (2020).

25. K. Ravet, M. Pilon, Copper and iron homeostasis in plants: The challenges of oxidative stress. *Antioxid. Redox Signal.* **19**, 919–932 (2013).

26. M. Pilon, The copper microRNAs. *New Phytol.* **213**, 1030–1035 (2017).

27. L. Zheng, N. Yamaji, K. Yokosho, J. F. Ma, YSL16 is a phloem-localized transporter of the copper-nicotianamine complex that is responsible for copper distribution in rice. *Plant Cell* **24,** 3767–3782 (2012).

28. C. Curie, G. Cassin, D. Couch, F. Divol, K. Higuchi, M. Le Jean, J. Misson, A. Schikora, P. Czernic, S. Mari, Metal movement within the plant: Contribution of nicotianamine and yellow stripe 1-like transporters. *Ann. Bot.* **103**, 1–11 (2009).

29. H. Sheng, Y. Jiang, M. R. Ishka, J. C. Chia, T. Dokuchayeva, Y. Kavulych, T. O. Zavodna, P. N. Mendoza, R. Huang, L. M. Smieshka, J. Miller, A. R. Woll, O. I. Terek, N. D. Romanyuk, M. Piñeros, Y. Zhou, O. K. Vatamaniuk, YSL3-mediated copper distribution is required for fertility, seed size and protein accumulation in Brachypodium. *Plant Physiol.* **186**, 655–676 (2021).

30. R. J. DiDonato, Jr., L. A. Roberts, T. Sanderson, R. B. Eisley, E. L. Walker, *Arabidopsis Yellow Stripe-Like2* (*YSL2*): A metal-regulated gene encoding a plasma membrane transporter of nicotianamine-metal complexes. *Plant J.* **39**, 403–414 (2004).

31. H. Wintz, T. Fox, Y. Y. Wu, V. Feng, W. Chen, H. S. Chang, T. Zhu, C. Vulpe, Expression profiles of Arabidopsis thaliana in mineral deficiencies reveal novel transporters involved in metal homeostasis. *J. Biol. Chem.* **278**, 47644–47653 (2003).

32. F. Deng, N. Yamaji, J. Xia, J. F. Ma, A member of the heavy metal P-type ATPase OsHMA5 is involved in xylem loading of copper in rice. *Plant Physiol.* **163**, 1353–1362 (2013).

33. M. Yuan, X. Li, J. Xiao, S. Wang, Molecular and functional analyses of COPT/Ctr-type copper transporter-like gene family in rice. *BMC Plant Biol.* **11**, 69 (2011).

34. J. Kropat, S. Tottey, R. P. Birkenbihl, N. Depège, P. Huijser, S. Merchant, A regulator of nutritional copper signaling in Chlamydomonas is an SBP domain protein that recognizes the GTAC core of copper response element. *Proc. Natl. Acad. Sci. U.S.A.* **102**, 18730–18735 (2005).

35. F. Sommer, J. Kropat, D. Malasarn, N. E. Grossoehme, X. Chen, D. P. Giedroc, S. S. Merchant, The CRR1 nutritional copper sensor in Chlamydomonas contains two distinct metal-responsive domains. *Plant Cell* **22**, 4098–4113 (2010).

36. H. Yamasaki, M. Hayashi, M. Fukazawa, Y. Kobayashi, T. Shikanai, SQUAMOSA promoter binding protein-like7 is a central regulator for copper homeostasis in Arabidopsis. *Plant Cell* **21**, 347–361 (2009).

37. M. Bernal, D. Casero, V. Singh, G. T. Wilson, A. Grande, H. Yang, S. C. Dodani, M. Pellegrini, P. Huijser, E. L. Connolly, S. S. Merchant, U. Krämer, Transcriptome sequencing identifies SPL7-regulated copper acquisition genes FRO4/FRO5 and the copper dependence of iron homeostasis in Arabidopsis. *Plant Cell* **24**, 738–761 (2012).

38. S. Lu, Q. Li, H. Wei, M. J. Chang, S. Tunlaya-Anukit, H. Kim, J. Liu, J. Song, Y. H. Sun, L. Yuan, T. F. Yeh, I. Peszlen, J. Ralph, R. R. Sederoff, V. L. Chiang, Ptr-miR397a is a negative regulator of

laccase genes affecting lignin content in Populus trichocarpa. *Proc. Natl. Acad. Sci. U.S.A.* **110**, 10848–10853 (2013).

39. C. Ma, S. Burd, A. Lers, miR408 is involved in abiotic stress responses in Arabidopsis. *Plant J.* **84**, 169–187 (2015).

40. Y. Zhao, S. Lin, Z. Qiu, D. Cao, J. Wen, X. Deng, X. Wang, J. Lin, X. Li, MicroRNA857 is involved in the regulation of secondary growth of vascular tissues in Arabidopsis. *Plant Physiol.* **169**, 2539–2552 (2015).

41. H. I. Jung, S. R. Gayomba, M. A. Rutzke, E. Craft, L. V. Kochian, O. K. Vatamaniuk, COPT6 is a plasma membrane transporter that functions in copper homeostasis in Arabidopsis and is a novel target of SQUAMOSA promoter-binding protein-like 7. *J. Biol. Chem.* **287**, 33252–33267 (2012).

42. M. Yuan, Z. Chu, X. Li, C. Xu, S. Wang, The bacterial pathogen *Xanthomonas oryzae* overcomes rice defenses by regulating host copper redistribution. *Plant Cell* **22**, 3164–3176 (2010).

43. E. Himelblau, R. M. Amasino, Delivering copper within plant cells. *Curr. Opin. Plant Biol.* **3**, 205–210 (2000).

44. E. Sudo, M. Itouga, K. Yoshida-Hatanaka, Y. Ono, H. Sakakibara, Gene expression and sensitivity in response to copper stress in rice leaves. *J. Exp. Bot.* **59**, 3465–3474 (2008).

45. S. Zhao, W. Hong, J. Wu, Y. Wang, S. Ji, S. Zhu, C. Wei, J. Zhang, Y. Li, A viral protein promotes host SAMS1 activity and ethylene production for the benefit of virus infection. *eLife* **6**, e27529 (2017).

46. B. Wu, J. S. Becker, Imaging techniques for elements and element species in plant science. *Metallomics* **4**, 403–416 (2012).

47. Q. Cai, L. Qiao, M. Wang, B. He, F. M. Lin, J. Palmquist, S. D. Huang, H. Jin, Plants send small RNAs in extracellular vesicles to fungal pathogen to silence virulence genes. *Science* **360**, 1126–1129 (2018).

48. Q. L. Wang, A. Z. Sun, S. T. Chen, L. S. Chen, F. Q. Guo, SPL6 represses signalling outputs of ER stress in control of panicle cell death in rice. *Nat. Plants* **4**, 280–288 (2018).

49. J. Wang, L. Zhou, H. Shi, M. Chern, H. Yu, H. Yi, M. He, J. Yin, X. Zhu, Y. Li, W. Li, J. Liu, J. Wang, X. Chen, H. Qing, Y. Wang, G. Liu, W. Wang, P. Li, X. Wu, L. Zhu, J. M. Zhou, P. C. Ronald, S. Li, J. Li, X. Chen, A single transcription factor promotes both yield and immunity in rice. *Science* **361**, 1026–1028 (2018).

50. H. Sun, X. Guo, X. Qi, F. Feng, X. Xie, Y. Zhang, Q. Zhao, SPL14/17 act downstream of strigolactone signalling to modulate rice root elongation in response to nitrate supply. *Plant J.* **106**, 649–660 (2021).

51. J. Li, B. Tang, Y. Li, C. Li, M. Guo, H. Chen, S. Han, J. Li, Q. Lou, W. Sun, P. Wang, H. Guo, W. Ye, Z. Zhang, H. Zhang, S. Yu, L. Zhang, Z. Li, Rice SPL10 positively regulates trichome development through expression of HL6 and auxin-related genes. *J. Integr. Plant Biol.* **63**, 1521–1537 (2021).

52. J. Yan, J. C. Chia, H. Sheng, H. I. Jung, T. O. Zavodna, L. Zhang, R. Huang, C. Jiao, E. J. Craft, Z. Fei, L. V. Kochian, O. K. Vatamaniuk, Arabidopsis pollen fertility requires the transcription factors CITF1 and SPL7 that regulate copper delivery to anthers and jasmonic acid synthesis. *Plant Cell* **29**, 3012–3029 (2017).

53. A. Y. Guo, Q. H. Zhu, X. Gu, S. Ge, J. Yang, J. Luo, Genome-wide identification and evolutionary analysis of the plant specific SBP-box transcription factor family. *Gene* **418**, 1–8 (2008).

54. R. Yang, P. Li, H. Mei, D. Wang, J. Sun, C. Yang, L. Hao, S. Cao, C. Chu, S. Hu, X. Song, X. Cao, Fine-tuning of miR528 accumulation modulates flowering time in rice. *Mol. Plant* **12**, 1103–1113 (2019).

55. J. Miao, D. Guo, J. Zhang, Q. Huang, G. Qin, X. Zhang, J. Wan, H. Gu, L.-J. Qu, Targeted mutagenesis in rice using CRISPR-Cas system. *Cell Res.* **23**, 1233–1236 (2013).

56. S. Fu, Y. Xu, C. Li, Y. Li, J. Wu, X. Zhou, Rice stripe virus interferes with S-acylation of remorin and induces its autophagic degradation to facilitate virus infection. *Mol. Plant* **11**, 269–287 (2018).

57. Q. Huang, Z. Mao, S. Li, J. Hu, Y. Zhu, A non-radioactive method for small RNA detection by Northern blotting. *Rice* **7**, 26 (2014).

58. J. P. Zhang, Y. Yu, Y. Z. Feng, Y. F. Zhou, F. Zhang, Y. W. Yang, M. Q. Lei, Y. C. Zhang, Y. Q. Chen, MiR408 regulates grain yield and photosynthesis via a phytocyanin protein. *Plant Physiol.* **175**, 1175–1185 (2017).

59. C. Liu, Y. Xin, L. Xu, Z. Cai, Y. Xue, Y. Liu, D. Xie, Y. Liu, Y. Qi, Arabidopsis ARGONAUTE 1 binds chromatin to promote gene transcription in response to hormones and stresses. *Dev. Cell* **44**, 348–361.e7 (2018).

60. C. A. Schneider, W. S. Rasband, K. W. Eliceiri, NIH Image to ImageJ: 25 years of image analysis. *Nat. Methods* **9**, 671–675 (2012).

61. W. Tang, W. A. Thompson, OsmiR528 enhances cold stress tolerance by repressing expression of stress response-related transcription factor genes in plant cells. *Curr. Genomics* **20**, 100–114 (2019).

62. Y. C. Zhang, R. R. He, J. P. Lian, Y. F. Zhou, F. Zhang, Q. F. Li, Y. Yu, Y. Z. Feng, Y. W. Yang, M. Q. Lei, H. He, Z. Zhang, Y. Q. Chen, OsmiR528 regulates rice-pollen intine formation by targeting an uclacyanin to influence flavonoid metabolism. *Proc. Natl. Acad. Sci. U.S.A.* **117**, 727–732 (2020).
